# Neural dynamics outside task-coding dimensions drive decision trajectories through transient amplification

**DOI:** 10.1101/2025.11.20.689599

**Published:** 2025-11-22

**Authors:** Ulises Pereira-Obilinovic, Kayvon Daie, Susu Chen, Karel Svoboda, Ran Darshan

**Affiliations:** 1 Allen Institute for Neural Dynamics, Seattle, WA, United States of America; 2 Janelia Research Campus, Howard Hughes Medical Institute, Ashburn, VA, USA; 3 Department of Physiology and Pharmacology, Faculty of Medicine, Sagol School of Neuroscience, The School of Physics and Astronomy, Tel Aviv University, Tel Aviv, Israel

## Abstract

Most behaviors involve neural dynamics in high-dimensional activity spaces. A common approach is to extract dimensions that capture task-related variability, such as those separating stimuli or choices, yielding low-dimensional, task-aligned neural activity subspaces (“coding dimensions”). However, whether these dimensions actively drive decisions or merely reflect underlying computations remains unclear. Moreover, neural activity outside these coding subspaces (“residual dimensions”) is often ignored, though it could also causally shape neural dynamics driving behavior. We developed a recurrent neural network model that fits population activity and uncovers the dynamic interactions between coding and residual subspaces on single trials. Applied to electrophysiological recordings from the anterior lateral motor cortex (ALM) and motor thalamus in mice performing a delayed response task, our model demonstrates that perturbations of residual dimensions reliably alter behavioral choices, whereas perturbations of the choice dimension, which strongly encodes the animal’s upcoming decision, are largely ineffective. These perturbation effects arise because residual dimensions drive transient amplification across an intermediate number of coding and residual dimensions (~10), before the dynamics collapse into discrete attractor states corresponding to the animal’s choice. By dissecting the low-dimensional variability underlying error trials, we find that it primarily shifts trajectories along residual dimensions, biasing single decisions. Residual activity in thalamus shapes cortical decision dynamics, implicating weakly selective thalamic populations in the emergence of cortical selectivity. Our findings challenge the conventional focus on low-dimensional coding subspaces as sufficient framework for understanding neural computations, demonstrating that dimensions previously considered task-irrelevant and accounting for little variance can have a critical role in driving behavior.

## Introduction

During decision-making, neural activity encodes task variables across large populations of neurons. Most studies of the neural dynamics underlying decisions have focused on the neural correlates of the task variables, such as those encoding sensory stimuli, choices, and actions (Gold & Shadlen 2007). Neurons encoding these decision variables are widespread across multiple brain regions (Steinmetz et al. 2019; Chen et al. 2024; Angelaki et al. 2025). The observation of neurons encoding decision variables does not establish causality. Although stimulating highly selective neurons can modulate behavioral performance in predictable ways (Salzman et al. 1990; Carrillo-Reid et al. 2019; Marshel et al. 2019; Robinson et al. 2020), emerging evidence suggests that decisions can be shaped by populations of neurons that do not explicitly code for the decision variables (Daie et al. 2023; Gauld et al. 2024; Bounds et al. 2024).

With the rise of large-scale neural recordings, the focus of studying neural dynamics has shifted from individual neurons to population-level activity. Applying dimensionality-reduction techniques to recordings of neural populations (Cunningham & Yu 2014; Pang et al. 2016; Kobak et al. 2016; Williams et al. 2018; Li et al. 2016; Pellegrino et al. 2024) has identified low-dimensional representations that capture decision-related variance (‘coding dimensions’), while discarding components that encode task variables only weakly (‘residual dimensions’). Each dimension represents a weighted combination of recorded neurons, and the coding dimensions typically align with task variables such as stimulus, choice, or outcome. These methods have provided insights into the evolution of neural trajectories and their relationship to decisions and behavior (Vyas et al. 2020; Gallego et al. 2017; Li et al. 2016; Steinemann et al. 2024; Monsalve-Mercado & Miller 2025).

Whether through statistical analyses that extract low-dimensional subspaces aligned with task variables (Mante et al. 2013; Kobak et al. 2016; Li et al. 2016), mechanistic circuit models of population dynamics (Wang 2002; Wong & Wang 2006; Machens et al. 2005; Wimmer et al. 2015), or data-driven dynamical models (T. D. Kim et al. 2023; Langdon & Engel 2025; Luo et al. 2025), the prevailing view is that neural dynamics causally related to decisions unfold largely within these task-coding dimensions. In this framework, activity along the coding dimensions is assumed to be self-sustaining and sufficient to generate decisions, while the remaining neural activity is typically disregarded. But just as tuning in single neurons does not imply causal relevance, it remains unclear whether the extracted coding dimensions causally shape decisions or simply correlate with them. Could the so-called *residual dimensions*, with minimal task encoding and potentially low variance, play a role in shaping neural dynamics and computation during decision-making?

To address this question, we developed a neural network framework that explicitly models neural activity by distinguishing task-coding from residual subspaces. The network is constrained to include all possible recurrent interactions between coding and residual dimensions, with coding dimensions optimized to represent key task variables (such as sample, choice, and response) and residual dimensions defined as orthogonal directions capturing the remaining variance. Unlike traditional dimensionality reduction methods, this approach enables direct modeling of the dynamic interactions between these two classes of dimensions at the single-trial level. In contrast to previous activity-constrained network models (Rajan et al. 2016; Finkelstein et al. 2021; DePasquale et al. 2023; Valente et al. 2022; C. M. Kim et al. 2023; Sourmpis et al. 2023; Pals et al. 2024; Sourmpis et al. 2024; Langdon & Engel 2025), our approach reduces the high-dimensional recurrent dynamics to population-level latent variables capturing activity along the coding and residual dimensions, and explicitly characterizes the interactions between these two sets of dimensions.

We applied our modeling framework to large-scale electrophysiological recordings from the anterior lateral motor cortex (ALM) and connected thalamus in mice performing a delayed-response decision-making task (Chen et al. 2024). Consistent with recent findings (Daie et al. 2023), we observed that perturbations along residual dimensions, which are orthogonal to the choice axis encoding decisions, were more effective at disrupting neural trajectories and altering decisions compared to perturbations along coding dimensions. In contrast to classical attractor models (Wang 2002; Wong & Wang 2006), which posit two dimensional dynamics arising from two competing choice-related populations that directly govern decision outcomes, our analyses revealed that neural dynamics operate within subspaces of intermediate dimensionality (~10 dimensions). This dimensionality is substantially higher than the low-dimensional coding subspace, but much smaller than the full population space. During decision formation, trajectories within these subspaces evolve through non-normal amplification, a regime in which interactions between nearly orthogonal activity patterns transiently amplify small deviations in neural trajectories (Goldman 2009; Murphy & Miller 2009), before they reliably converge onto discrete attractors corresponding to decision outcomes (Inagaki et al. 2019; Oña-Jodar et al. 2024). As a result, even small perturbations along residual dimensions, which are dominated by neurons with low firing rates and weak task selectivity, can transiently grow and redirect trajectories within the coding subspace. Our findings challenge the prevailing view that low dimensional coding subspaces alone capture the neural computations underlying behavior.

## Results

### Coding and residual dimensions in activity-trained networks

**Figure 1:**
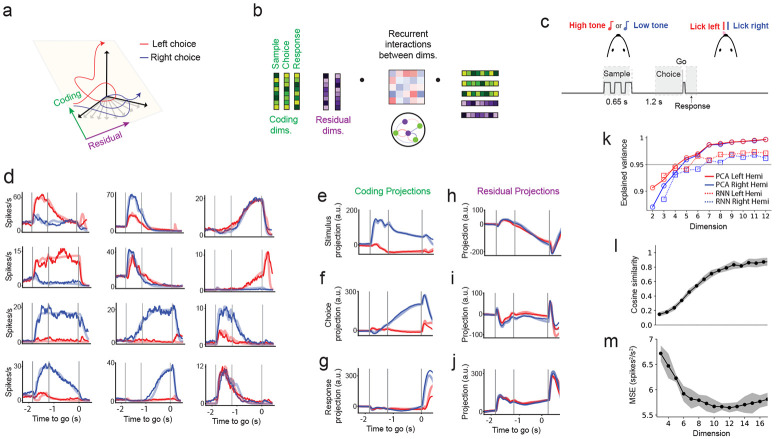
Coding and residual dimensions in activity-trained networks in a memory-guided decision-making task. (a) Neural trajectories during decision-making for two trial types. (b) Network connectivity structure. Connectivity is defined by all possible combinations of coding and residual dimensions. The vectors representing these dimensions and their recurrent interactions are optimized to maximize the encoding of behavioral variables, while fitting neural activity. (c) Schematic of the memory-guided decision-making task. Auditory stimuli (high tone, low tone) presented during the sample epoch instruct a directional movement (left, right), after a 1.2 s delay epoch. (d) Trial-averaged activity for correct left and right trials (right hemisphere). Dark lines, neural recordings; light lines, model predictions. Total number of dimension, 10. (e-j) Projections of neural activity onto coding and residual dimensions over time. Coding projections align with task variables (stimulus, choice, response). (k) Explained variance of neural activity from PCA (circles, solid lines) and RNN (squares, dashed lines) as a function of the number of dimensions. The gray line marks 95% explained variance. (l) Average cosine similarity between the coding dimensions extracted from the network fit and those derived from the data, computed across five independent training sets using 80% of the trials to estimate trial-averaged activity and train the network, shown as a function of model dimensionality. (m) Cross-validated mean squared error (MSE) between the network’s firing rates and the trial-averaged activity computed on the held-out 20% of trials in each subset. Error bars indicate the standard error across folds (see Methods). In (l) and (m) the traces are averaged across hemispheres (see Fig. S1).

To investigate how residual dimensions contribute to neural computations, we developed a recurrent neural network (RNN) model trained to simultaneously fit large-scale neural recordings and uncover latent subspaces associated with behavior. Specifically, we sought to distinguish between coding dimensions—subspaces aligned with task-relevant variables such as sensory stimuli, choices, and motor outputs—and residual dimensions, which are orthogonal to the coding subspace but might still contribute to the RNN’s ability to reproduce the observed neural dynamics (Fig. 1a). This modeling framework enables us to move beyond conventional dimensionality reduction methods and examine how structured variability outside task-related dimensions supports neural computation.

We modeled the firing-rate dynamics of each recorded neuron, $r_{i}(t)$, using a nonlinear recurrent neural network of N units, whose activity evolves under recurrent interactions specified by the connectivity matrix J (Methods, Eq. (3)). Each neuron received external input, representing task events such as the presentation of a sample stimulus and a go cue (see the description of the decision-making task, below). Neuronal dynamics were governed by integration time constant of 40 ms, consistent with cortical membrane time scales. Each neuron transformed its net input through a nonlinear transfer function, $\phi_{i}(x)$, with heterogeneous saturation levels across the population (see Methods, Eq. (4)). Importantly, each neuron in the model corresponds to a single neuron from the neural recordings (see below).

In our modeling framework, we structured the RNN so that its recurrent connectivity explicitly captured interactions between coding and residual dimensions. Specifically, we constrained the connectivity matrix between two neurons (i,j∈{1…N}) to be low-rank (Mastrogiuseppe & Ostojic 2018)

(1) $$J_{ij}=\sum_{l=1}^{P}\sum_{l^{\prime }=1}^{P}A_{ll^{\prime }}u_{i}^{l}u_{j}^{l^{\prime }}.$$

The low-rank structure was defined by a set of orthonormal vectors: ${\left\{{\vec{u}}^{l}\right\}}_{l=1}^{C}$ spanning the C coding dimensions (Methods and see below) and ${\left\{{\vec{u}}^{l}\right\}}_{l=C+1}^{P}$ spanning the residual subspace, which is orthogonal to the coding subspace.

The interaction matrix A governs how population dynamics within and across these subspaces interact (Fig. 1b). This formulation can capture a broad range of population dynamics, including fixed-point attractors (Hopfield 1982), sequential activity (Kleinfeld 1986; Sompolinsky & Kanter 1986; Gillett et al. 2020; Gillett & Brunel 2024), and continuous attractors (Darshan & Rivkind 2022; Khona & Fiete 2022). By explicitly parameterizing interactions between coding and residual dimensions in the interaction matrix A, we are able to dissect the interactions between task-coding and residual components of the network activity.

Projection of the neural trajectories, $\vec{r}(t)$, onto the coding and residual subspaces define the latent variables in our model

(2) $$m_{l}(t)=\sum_{i=1}^{N}u_{i}^{l}r_{i}(t).$$

Thus, by projecting the network dynamics onto the coding and residual subspaces, we obtained a reduced description of the population activity in terms of latent variables ${\left\{m_{l}\right\}}_{l=1}^{P}$, which correspond to the projection of firing rates onto the low-rank basis (see Methods, Eq. (11)). The latent dynamics evolve according to a P-dimensional nonlinear dynamical system that captures how population activity evolves within the coding and residual subspaces. This formulation enables us to assess the extent to which residual dynamics shape trajectories along coding dimensions.

### Training on large-scale neural recordings from motor cortex during decision-making

We applied our modeling framework to large-scale electrophysiological recordings spanning cortical and subcortical structures in mice performing a memory-guided decision-making task (Chen et al. 2024) (Fig. 1c; see Methods). On each trial, a brief auditory cue (either high or low frequency) signaled a left or right lick response following a 1.2-second delay. This task requires the animal to transform a transient sensory input into a persistent motor plan maintained over the delay period (Li et al. 2016).

To identify the C coding dimensions, which is the subspace aligned with task-relevant variables, we averaged neural activity across trials to obtain population trajectories, where each time point is a point in an N-dimensional neural space (Vyas et al. 2020). We used dimensionality reduction method (linear discriminant analysis, LDA; Methods) to find the directions that best separated behavioral conditions, such as left versus right choices. This approach isolates interpretable task-relevant dimensions of population activity (Li et al. 2016; Inagaki et al. 2019). Consistent with previous studies, we focused on three dimensions: sample, choice, and response (C=3). The sample dimension captured stimulus-evoked activity in the first 600 ms of the trial; the choice dimension separated left and right trials during the delay period (600 ms before the go cue); and the response dimension distinguished left and right licks 350 ms after the go cue (Li et al. 2016; Chen et al. 2024) (Fig. 1c). Together, these dimensions explain approximately 20% (see Methods) of the population variance in ALM and capture key computational epochs: stimulus encoding, decision formation, and motor output. We focused on these dimensions because they reflect the core computational demands of the task: sensory integration (Finkelstein et al. 2021), choice maintenance (Li et al. 2016; Chen et al. 2021; Wang et al. 2021), and motor execution (Inagaki et al. 2022; Chen et al. 2024). Among these dimensions, the choice dimension has received particular attention (Li et al. 2016; Finkelstein et al. 2021; Inagaki et al. 2022; Daie et al. 2023; Wang et al. 2021; Chen et al. 2024), as it is strongly correlated with the animal’s upcoming decision (Inagaki et al. 2019) and remains stable throughout the delay period (Li et al. 2016; Inagaki et al. 2018; Daie et al. 2023).

The remaining R=P−C dimensions define the residual subspace. These residual dimensions are orthogonal to the coding dimensions and are not designed to align with task variables. Rather than assigning them predefined functional roles, we allowed the RNN to discover how they support the observed dynamics throughout the training procedure. The training of the RNN thus jointly identifies the low-dimensional subspace spanned by both coding and residual dimensions, the interaction structure among these dimensions, and the recurrent dynamics that best reproduce the neural data.

We first focused on the anterior lateral motor cortex (ALM), a dorsal cortical area required for maintaining choice-related activity (Guo et al. 2014). We trained the network to reproduce condition-averaged activity from 7,884 neurons (3,640 in the left hemisphere, 4,244 in the right) during correct (hit) left and right trials (Fig. 1d). During the sample period (Fig. 1c), the network received two distinct inputs corresponding to left and right auditory cues that were projected onto both task-coding and residual dimensions ($I_{i}^{\text{task}}(t)$ in Eq. (7,8), Methods). The alignment of these inputs was optimized through training (see Methods). Similar input were received by the network for the go cue (Eq. (7), Methods).

Unlike dimensionality reduction methods that extract low-dimensional structure passively, the network was required to integrate transient task-related sensory information, maintain selective activity during the delay, and transform this delay activity into response-selective activity after the go cue, while matching the recorded neural dynamics and aligning the coding subspace to that extracted from data. This approach allowed us to study a recurrent network model that simultaneously fit both the neural activity and the latent dynamics. Projections onto task-coding dimensions recapitulated known features of ALM activity (Fig. 1e-g), including ramping along the choice axis during the delay period (Li et al. 2016; Chen et al. 2024) (Fig. 1f). Residual dimensions exhibited little selectivity for left versus right conditions (Fig. 1h-j).

Consistent with previous findings, trial-averaged neural activity resides in a low-dimensional subspace. Principal component analysis (PCA) of the trial-averaged population responses revealed that over 95% of the variance was captured by the first five components (Fig. 1k). Our trained low-rank models reflected a similar low-dimensional structure.To quantify this, we first fixed the model’s three components to align with empirically estimated coding dimensions and trained only the recurrent interaction matrix. This configuration explained 78% of the variance, much higher than the 20% explained without training the interaction matrix (see above). Next, we introduced a soft alignment constraint, allowing the coding components and interaction matrix to be co-optimized, while penalizing deviations from the data-derived coding dimensions (Methods). This increased the explained variance to nearly 90%.

Despite this improvement, models constrained to use only the coding dimensions exhibited poor alignment with the empirical task axes derived from linear discriminant analysis (LDA), yielding an average cosine similarity of 0.15 (~ 80° alignment; Fig. 1l). This suggested that additional dimensions beyond the coding subspace were needed to reproduce task-relevant dynamics in addition to the recorded single neuron dynamics. We therefore retained the soft alignment and incrementally incorporated additional residual dimensions. Adding these dimensions yielded only a modest increase in explained variance, from 90% to 97% as the total dimensionality increased from 3 to 12 (Fig. 1k), but this small variance gain was accompanied by a substantial improvement in alignment with the empirical task axes (Fig. 1l and Fig. S1a). Cosine similarity increased sharply and plateaued around 12 total dimensions. Thus, a combination of coding and residual dimensions are needed in the network model to capture both the task-relevant and single neuron dynamics dynamics with high fidelity.

**Figure 2:**
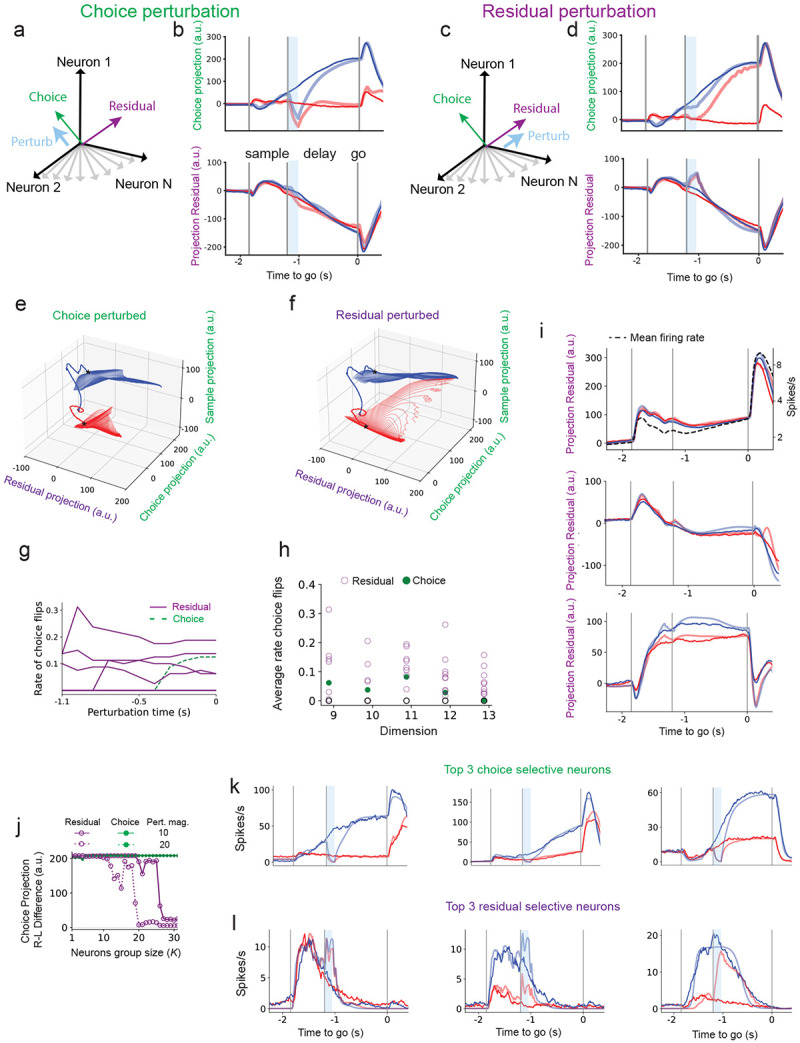
Choice-aligned perturbations preserve choices while residual-aligned perturbations disrupt them. (a) Schematic illustrating a 150 ms perturbation in the choice direction at the onset of the delay period (blue arrow). (b) Projections of neural activity onto the choice (top) and residual (bottom) dimensions for perturbations aligned with the choice. Blue bar indicates the perturbation period. (c-d) Same as (a-b), but for perturbations aligned with the residual dimension. (e-f) Network responses for perturbations with magnitudes ranging from −10 to 10 applied along the choice dimension (e) and an influential residual dimension (f), visualized in the 3D subspace defined by choice, sample, and residual axes. Trajectories are shown until the go cue. A black star indicates the start of the delay period.(g) Rate of choice flips for 11 distinct 100 ms perturbation intervals applied during the delay period, across 10 network dimensions. Each dimension was tested with perturbation magnitudes from −15 to 15 (see Methods). (h) Delay-period average choice-flip rates from (g), summarized across networks trained with 9 to 13 dimensions. Black circles indicate the average effect of 10 random perturbations orthogonal to both the coding and residual dimensions, matched in perturbation magnitude. (i) Dynamics of projections for three additional influential dimensions beyond the one shown in (d). Solid lines depict projections from neural recordings; lighter lines show model fits. Dashed line in top panel shows the mean firing rate across neurons and left and right trials. (j) Absolute difference in the choice-axis projection between left- and right-choice trials, averaged over the 100 ms preceding the go cue, as a function of perturbed group size. For each group size K (number of neurons in the perturbed set), we perturb the top-K neurons ranked by selectivity, either the top-K choice-selective or the top-K residual-selective neurons, where rankings are defined in the Methods. Perturbations are applied during the first 100 ms of the delay period as in (a-d). (k) Responses of the top 3 choice-selective neurons to perturbations targeting the top 30 choice-selective neurons. (l) Same as (k), but for the top 3 residual-selective neurons when targeting the top 30 residual-selective neurons.

**Figure 3:**
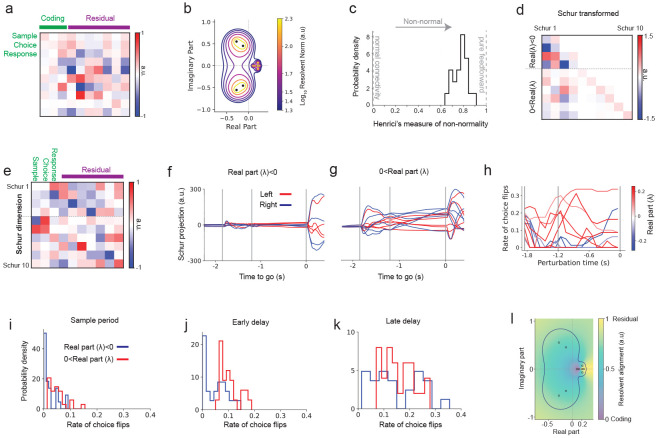
Non-normal interactions between coding and residual dimensions shape decision trajectories. (a) Interaction matrix A for the network shown in Fig. 2, with latent dimensionality P=10. (b) Eigenvalue spectrum and pseudospectra of the interaction matrix (Trefethen & Embree 2020). Contour lines correspond to resolvent norms of 5 × 10^−2^, 4 × 10^−2^, 3 × 10^−2^, 2 × 10^−2^, 1 × 10^−2^, and 5 × 10^−3^ (Methods). (c) Normalized Henrici index (see Methods) across 40 trained networks with latent dimensionality ranging from P=9 to P=16. (d) Schur form (lower-triangular matrix) of the interaction matrix. Diagonal entries indicate the real parts of eigenvalues; red and blue indicate Schur dimensions with positive and negative real parts, respectively. Dashed line separates the two groups. (e) Unitary matrix defining the orthonormal Schur basis. Rows correspond to Schur vectors; the dashed line separates dimensions associated with positive versus negative real eigenvalues. (f, g) Network activity projected onto Schur dimensions associated with negative (f) and positive (g) real eigenvalues for left (red) and right (blue) choice trials. (h) Rate of choice flips for perturbations applied to each Schur dimension across 18 non-overlapping 100 ms windows spanning the sample and delay periods. Each dimension was tested with perturbation magnitudes from −15 to 15. Line colors indicate the real part of the associated eigenvalue. (i–k) Mean rate of choice flips for Schur dimensions with negative (blue) and positive (red) real eigenvalues, averaged across perturbations applied during the sample period (i), early delay (−1.2 to −0.6 s; j), and late delay (−0.6 to 0 s; k). (l) Alignment of the resolvent to the coding and residual subspaces. Alignment between the dominant right-singular vector of the resolvent and the residual subspace (see Methods). Yellow indicate regions in the complex plane where the most amplifiable input directions align strongly with residual subspace, whereas purple regions indicate strong alignment with the coding subspace. The lowest resolvent contour from panel b is overlaid for visual correspondence of both panels.

**Figure 4:**
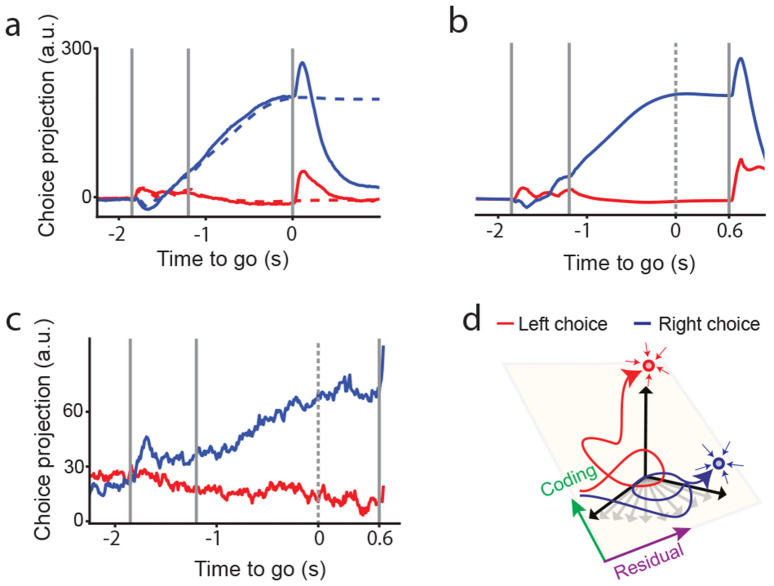
Network delay dynamics ends in two attractor states selective for left and right choices. (a) Choice projection from the network when the go cue is withheld (dashed line) and from neural recordings for 1.2s delay trials (solid line). (b) Choice projection from the network when the go cue is presented at 1.8s after the sample period, simulating a catch trial. The original go cue at 1.2s is indicated by a dashed line. (c) Trial-averaged activity of 2529 recorded ALM neurons projected onto the choice dimension for catch trials with a 1.8s delay. (d) Schematic of selective high-dimensional delay activity converging into distinct attractor states for left and right choices.

Cross-validated loss was minimized in models with 10–12 dimensions (Fig. 1m, comprising 3 coding and 7–9 residual dimensions). Based on this, we focus our subsequent analyses on a 10-dimensional model (Figs. 2-4), which provided the best overall fit while maximizing alignment with data-derived coding axes (Fig. 1l, m; Fig. S1a,b). Notably, all the results below were robust across intermediate dimensionalities (P=9−13), random seeds, and hemispheres.

Having established a network model that accurately captures both single-neuron activity and latent dynamics along the coding and residual subspaces, we next asked: what computational role do these residual dimensions play in shaping decision-related dynamics in this task?

### Choice dimension aligned perturbations preserve choice while residual aligned perturbations disrupt it

We next studied the trained model through perturbing its activity along the coding and residual dimensions. We found that perturbations along the choice dimension (Fig. 2a), despite its strong correlation with behavior, had little effect on the final decision (Fig. 2b; Fig. S2 a-c). In contrast, perturbing a subset of residual dimensions, which have minimal choice selectivity, consistently flipped the choice projection from one outcome to the other (e.g., left to right in Fig. 2d; Fig. S2 d-i). These effects were robust across hemispheres (Fig. S2j-n), mirroring findings from two-photon perturbation experiments (Daie et al. 2023).

Projecting the network dynamics onto the sample, choice, and residual dimensions revealed that perturbations along the choice dimension caused only transient deviations that decayed back to the unperturbed trajectory (Fig. 2e). In contrast, sufficiently strong perturbations along few of the residual dimensions triggered large, nonlinear excursions, flipping the trajectory from one end point to the other (Fig. 2f). As the projection on choice is highly correlated with the animal’s choice, we consider such a flip at the end point of the trajectory at the end of the delay period as a flipped decision (Inagaki et al. 2019).

Extensive analysis of the networks response to such perturbations (see Methods) revealed that certain residual dimensions, when perturbed during the delay period, consistently showed a pronounced likelihood of inducing switches in choice behavior (Fig. 1, network trained on data from the right hemisphere; Fig. S2n similar results for data from the left hemisphere). We term these dimensions influential residual dimensions. In contrast, the choice dimension exhibits resistance to perturbations early in the delay period but becomes increasingly sensitive later on (Fig.S2n). This temporal progression in sensitivity is consistently observed across multiple trained networks and different numbers of residual dimensions, R (Fig. 2h). Random perturbations constructed to lie in the subspace orthogonal to the coding and residual dimensions had no effect on the network’s choice for comparable perturbation magnitudes (Fig. 2h and Fig. S2o, black markers), indicating that coding and residual dimensions are causal axes of the dynamics. Overall, our analyses demonstrate that a few of the residual dimensions are generally more influential in perturbing choice than the choice dimension, which only becomes sensitive later in the delay period (Fig. 2g and Fig. S2n). Notably, these influential residual dimensions account individually for only 1–4% of the total population variance.

Do residual dimensions align with known decision-related signals? Some of the influential residual dimensions correlate with population activity patterns previously associated with decision-making, such as ramping activity (Tanji & Evarts 1976; Churchland et al. 2010; Guo et al. 2014),(Fig. 2d, bottom) or the mean activity across both trial types (Fig. 2i, top). However, others show no clear correspondence with known task-related signals (Fig. 2i, middle and bottom). Moreover, while some influential residual dimensions exhibit detectable choice selectivity, this selectivity is typically very weak compared with the coding dimensions (Fig. 1l). These observations suggest that a dimension’s influence on choice is not necessarily tied to its alignment with task-evoked activity or choice selectivity.

Current experimental manipulations do not directly target specific dimensions in state space; they act on subsets of neurons (Marshel et al. 2019; Daie et al. 2021; Adesnik & Abdeladim 2021; Daie et al. 2023). Accordingly, we perturbed the top-K neurons most aligned with either the choice dimension or an influential residual dimension to test whether few-cell perturbations disrupt choice. Perturbing approximately 20–30 of the most residual-selective neurons abolished the left–right separation on the choice dimension flipping the network’s choice (Fig. 2j). By contrast, perturbing the top choice-selective neurons transiently reduced choice selectivity during the 100-ms perturbation, but it rebounded once the input ceased (Fig. 2j-k; See also Fig. S2r-t for opposite-sign excitatory perturbations). Consistent with perturbations targeting specific dimensions (Fig. 2d,f), perturbing residual-selective neurons effectively disrupted choice (Fig. 2j,l), despite choice-selective neurons having higher mean firing rates during the delay period (14 vs. 4 spikes/s); see Fig. S2p vs. q, continuous traces) and substantially stronger delay-period choice tuning (15 vs. 0.3 spikes/s), right–minus–left; dashed traces in Fig. S2p vs. q). These results indicate that neurons with weak selectivity can nonetheless exert strong causal influence on choice, as perturbations to these neurons are amplified through the network’s dynamics.

Together, these findings are consistent with recent work showing that weakly selective dimensions can causally control transitions between decision states while the choice dimension remains robust under perturbation (Daie et al. 2023).

### Non-normal interactions between coding and residual dimensions shape decision trajectories

Next, we sought to uncover the neural mechanisms through which residual dimensions exert causal control over transitions between decision states. To examine how these dimensions shape the underlying dynamics, we analyzed the interaction matrix, A, within our trained recurrent neural network. These interactions describe how latent variables influence each other over time (Eq. (11)). While the interaction matrix alone does not entirely define the dynamics due to nonlinearities, it organizes the flow of neural trajectories during decision-making.

We discovered strong, asymmetric interactions between coding and residual dimensions in the trained network (Fig. 3a). This finding implies that decision-making arises not solely from task-aligned neural signals– as typically assumed in models of decision-making (Wang 2002; Inagaki et al. 2019)– but also critically depends on interactions with dimensions traditionally considered irrelevant.

In the trained network, perturbations to a few of the residual dimensions at the beginning of the delay period got amplified and influenced the rate of choice flips later during the delay period. To study the mechanisms underlying such effect, we analyzed the interactions matrix. Transient dynamics can arise from non-normal interactions (Ganguli et al. 2008; Goldman 2009; Murphy & Miller 2009; Hennequin et al. 2012). A non-normal connectivity matrix describes a network where activity can be temporarily amplified due to asymmetries in how the different dimensions influence each other, even if the overall activity eventually settles down. This means that certain patterns of input can trigger strong, transient responses that would not occur in a network with symmetric (normal) connectivity.

To assess the degree of non-normality of the interactions, we utilized pseudospectra analysis (Trefethen & Embree 2020), a mathematical method that expands upon eigenvalue analysis by quantifying the network’s sensitivity to perturbations. Standard eigenvalue analysis identifies directions in neural state space along which activity grows (positive eigenvalues) or fades (negative eigenvalues). However, this approach can miss important effects in networks with asymmetric connections, where the eigenvalues can be highly sensitive to small variations in the interactions. Pseudospectral analysis (see Methods) reveals this hidden sensitivity and can identify non-normal interactions. For networks with normal interactions, the pseudospectra analysis shows isolated patterns. But in our models, the patterns were broad and overlapping, especially around areas linked to growing activity (positive eigenvalues), which is a clear signs of strong asymmetric and non-normal interactions (Fig. 3b; Fig. S3).

Another way to quantify the amount of non-normality of the interactions is given by the Henrici index (Trefethen & Embree 2020; Asllani et al. 2018) (Methods, Eq. (12)). This measure quantifies how far our interaction matrix deviate from a normal connectivity. It is zero for perfectly normal matrices, such as in thus typically assumed classical decision-making models (Wang 2002; Wong & Wang 2006), and grows to a maximum number of 1 when the network is strictly non-normal (Methods, Eq. (12)). The trained networks exhibited consistently high non-normality (mean normalized Henrici index across trained networks of 0.78; Fig. 3c).

To further dissect these structured interactions, we performed a Schur decomposition (Methods), which simplifies the interaction matrix into two distinct components: normal (symmetric) recurrent interactions and (non-normal) asymmetric interactions (Golub & Van Loan 2013). This decomposition provides intuitive insight into how transient neural activity emerges from non-normal amplification (Goldman 2009; Murphy & Miller 2009; Hennequin et al. 2012) (Fig. 3d,e). The Schur decomposition identified two distinct groups: stable (negative eigenvalues) and transiently amplifying (positive eigenvalues) dimensions. The stable dimensions are characterized by robust motor-execution period activity (Fig. 3f), while the transiently amplifying dimensions are active predominantly early in the delay period (Fig. 3g). Thus, neural activity dynamically balances transient amplification essential for decision formation with stable integration of the go cue needed for motor actions.

To directly assess the functional importance of these Schur dimensions we applied targeted perturbations to the trained model at different times of the task. Perturbations to transiently amplifying Schur dimensions (positive eigenvalues) strongly impacted decision outcomes early in the delay period (Fig. 3h, red curves), underscoring their causal role in shaping decisions. Conversely, perturbations of stable Schur dimensions (negative eigenvalues; Fig. 3h, blue curves) had pronounced effects mainly towards the go cue, aligning with action initiation (Fig. 3h-k). Critically, the differential timing of these perturbation effects highlights the transient, high-dimensional nature of neural dynamics underlying decision-making. Thus, transient amplification and stable integration of the go cue dynamically interact. Since the Schur decomposition expresses each dimension as a linear combination of coding and residual dimensions (Fig. 3e), residual dimensions play a critical role despite their minimal correlation with task variables.

To assess the role of residual dimensions in non-normal amplification, we identified the latent dimensions that the network transiently amplifies the most and examined how they align with coding and residual subspaces in the fitted networks (Methods). Regions of strongest amplification, located near eigenvalues with positive real parts and reflecting shared non-normal interactions among modes (Fig. 3b), consistently aligned with residual dimensions (Fig. 3l, compare with Fig. 3b). These eigenvalues correspond to Schur dimensions that exert strong effects on flipping the choice (Fig. 3h). These results show that the strongest non-normal transient amplification in latent space, induced by the interaction matrix A, predominantly unfolds along residual directions.

Our findings that the dynamics in the trained model is transient, of high dimensions (~10), and is driven by a non-normal interaction between the coding and the residual dimensions is robust and similar results were obtained across different trained networks (Fig. 3c, i-k), different dimensions (Fig. S3a-e) and datasets obtained from different hemispheres (Fig. S3f). These results illustrate a critical distinction between dimensions optimal for decoding decisions and those dynamically influential in controlling neural trajectories. Although the choice dimension effectively encodes decision outcomes, dynamically influential dimensions have distinct alignments, emphasizing the role of transient non-normal residual dynamics in shaping decision trajectories.

### Network delay transient dynamics ends in two attractor states selective for left and right choices

Our analysis showed that the network dynamics transiently evolves in ~10 dimensions during the delay period. This contrasts sharply with the two-dimensional dynamics predicted by classical decision-making models based on attractor dynamics (Wang 2002; Wong & Wang 2006). However, it is consistent with recent analyses of targeted optogenetic perturbations in ALM (Daie et al. 2023).

To test if we can reconcile these two perspectives, we examined the dynamics of the trained network at the end of the delay period. Specifically, we removed the go cue from the model after training. When the go cue is withheld, the network settles into one of two stable attractor states, each representing a distinct choice (Fig. 4a). This result can be further verified through analysis of catch trials. In the data, in a subset of the trials the go cue was delayed from 1.2s to 1.8s. Simulating these experimental catch trials using our model reveals that by 1.2s, the network has already reached a choice-selective attractor. The late go cue at 1.8s collapses the selective attractor states back to a non-selective state (Fig. 4b). Consistent with the network’s predicted dynamics, projections of the neural recordings during the experimental catch trials onto the choice dimension show that by 1.2s, activity in catch trials has stabilized (Fig. 4c). To assess the dynamical stability of the fixed points, we computed the eigenvalues of the Jacobian at the choice-selective attractors (see Methods) and found that the eigenvalue with the largest real part was negative across trained networks (Fig. S4a), confirming their stability. Our results were also replicated in networks trained on recordings from the left hemisphere (Fig. S4b).

To conclude, our analysis reveals that the dynamics of the trained networks reliably follow two distinct high-dimensional trajectories encompassing coding and residual dimensions. These trajectories transiently align with different dimensions in activity space before settling into selective attractor states corresponding to the two choices. During the delay, the network dynamics evolve along these high-dimensional transient paths, ultimately converging into distinct attractors for left versus right choices (Fig. 3d). Thus, the obtained dynamics reconcile the strong feedforward delay dynamics observed in Daie et al. 2023 with the attractor dynamics observed in ALM in studies where the delay period is randomized (Inagaki et al. 2019; Oña-Jodar et al. 2024) (see Discussion).

### Single-trial variability as a low-dimensional perturbation of the trial-average dynamics

**Figure 5:**
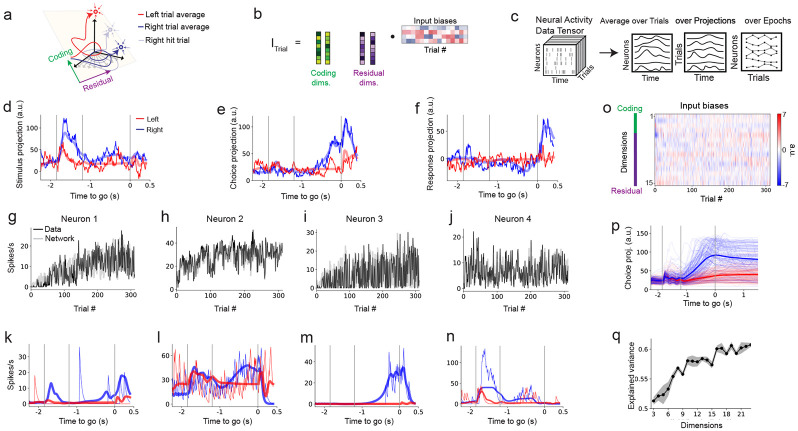
Fitting single-trial dynamics as low-dimensional perturbations from trial-averaged trajectories. (a) Schematic of trial-averaged trajectories for left- and right-hit trials (solid red and blue lines, respectively), and a single right-hit trial (lighter blue line). (b) Schematic of low-dimensional, trial-dependent input biases. Biases aligned with coding and residual dimensions are linearly combined to generate input currents that vary across trials. (c) Both the recurrent connectivity and trial-specific input biases (Eqs. (25, 26)) are optimized to fit the condition-averaged trajectories, their projections along coding dimensions, and the temporal average activity across the pre-sample, sample, delay, and response epochs (see Methods). (d–f) Single-trial dynamics projected onto the stimulus, choice, and response dimensions for left (red) and right (blue) trials. Solid lines represent recorded data; lighter lines show the corresponding network fits. (g–j) Time-averaged activity across trials for four example neurons. Black lines: data; gray lines: network fits. (k–n) Single-trial activity for the same neurons shown in (g–j) and the same trials shown in (d–f). Thin solid lines: convolved spike trains; thick lines: network fits. (o) Trial bias matrix after training, with P=15 rows and columns corresponding to individual trials. (p) Single-trial network trajectories projected onto the choice dimensions for correct left (red) and right (blue) trials when the go cue withheld. Thin lines: single-trial trajectories; thick lines: condition-averaged trajectory. (q) Explained variance on the test set across models of different dimensionalities P.

What role do residual dimensions play in shaping individual decisions? To investigate this, we incorporate in our model single-trial fluctuations as low-dimensional perturbations of the trial-average dynamics (Fig. 5a). We model trial-to-trial variability as changes in low-dimensional input biases to our recurrent network (Fig. 5b; see Methods Eq. (26)). The recurrent dynamics are held constant across trials, while the input bias varies from trial to trial and are also jointly trained (see Methods). Our model represents single-trial trajectories as perturbations of the trial-averaged low-dimensional flow field (Fig. 5a), capturing deviations around the condition-averaged dynamics. These input biases act as slow, across-trial modulations of within-trial dynamics (slower than the single-trial timescale), and may be interpreted as a proxy for modulatory inputs, e.g., from unobserved regions or neuromodulatory effects, that drift slowly and are not directly tied to task variables.

We applied this framework to ALM recordings on a session with more than 100 simultaneously recorded neurons. Following training, in addition to the recurrent connectivity we obtain the fluctuations in the trial biases that perturb the average trajectory on a trial-by-trial basis (Fig. 5o; see Methods, Eq. (26)). Importantly, these fluctuations lie in a subspace spanning both coding and residual dimensions (Fig. 5o).

We trained the network by simulating multiple trials and constraining it to match several summary statistics of the neural activity tensor (Fig. 5c). These included: the condition-averaged activity across trials, trial-averaged projections onto task-relevant coding dimensions (stimulus, choice, response; Fig. 5d-f), and time-averages computed within each of the task epochs (sample, delay, response; Fig. 5g-j). This strategy allows rate-based models to be trained on summary statistics extracted from single-trial spiking activity. Training on these summary statistics emphasizes shared structure across neurons and trials, enabling robust learning of stable latent dynamics and interpretable low-dimensional variability (Pellegrino et al. 2024).

As the network was trained to reproduce only summary statistics of the neural activity tensor, it was not designed to match the single-trial dynamics of individual neurons. The model nonetheless reproduces various features of the temporal dynamics at the single-neuron level, within and across trials (Fig. 5g-n). Importantly, single-trial activity exhibits non-stationary dynamics across trials, such as slow ramping of neural responses across trials, throughout the session (Fig. 5g-i). Such trial-dependent structure in the neural activity is captured by our method but would be missed by approaches that assume independent and identically distributed fluctuations across trials.

With the go cue withheld, the network fit to single trials often exhibits two choice-selective attractors, consistent with the trial-averaged model (Fig. 4; Fig. 5p). In this setting, trial-dependent input biases modify the flow field of the network dynamics, altering the local velocity of neural trajectories (Eq. (26)). These changes lead to trial-to-trial variations in the velocity of the choice projection uptake, as well as shifts in the location and stability of the attractors (Fig. 5p).

The test-set explained variance obtained through cross-validation saturates around 60% for a network dimensionality of about P=15 (Fig. 5q), which is slightly higher than the dimensionality inferred from models fitted to condition-averaged data (P=10−12).

Overall, these results demonstrate that the model effectively captures the shared low-dimensional structure across trials.

### Residual dimensions bias single-trial decisions

To study how residual dimensions could bias single-trial decisions we focused on analyzing miss trials, instances in which animals made incorrect choices by selecting the lick-port opposite to the stimulus cue. These trials provide a window into the sources of variability that can alter decision outcomes on a single-trial basis. Based on our previous findings using networks trained on condition-averaged activity, we hypothesized that such errors arise from trial-by-trial fluctuations along residual dimensions, which, while orthogonal to the task-coding subspace, exert a strong influence on the final choice.

**Figure 6:**
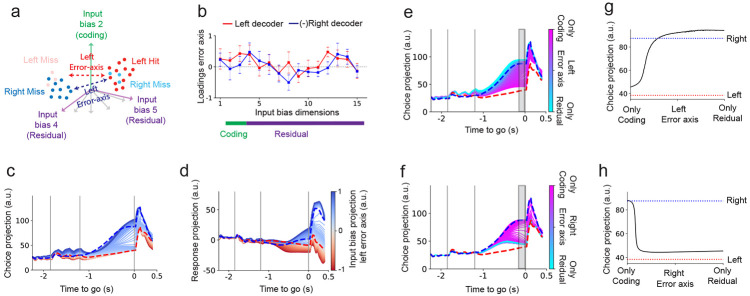
Residual dimensions bias single-trial decisions. (a) Schematic representation of the input bias space. Each trial is represented as a point corresponding to the fitted input bias (see Methods). The left and right error axes are defined as directions within the input bias space that optimally differentiate miss from hit trials for left and right conditions, respectively. (b) Error axis loadings for classifying hit versus miss trials. The first three components correspond to stimulus, choice, and response dimensions, while the remaining components span the residual subspace. Right-trial weights are shown with inverted signs for clarity. Error bars represent the standard deviation computed via bootstrapping (1000 iterations with 80% of trials). (c, d) Network projections onto the choice (c) and response (d) dimensions after systematic shifts of the input bias along the left error axis during left trials. Blue and red dashed lines indicate network fit to the trial-averaged correct left and right trials, respectively. (e) Trajectories in the choice projection for input biases of fixed magnitude that produce error-like left trials (i.e., trials whose choice projections approach the trial-averaged trajectory of right correct trials). The same perturbation magnitude is used while continuously changing the composition of the left error axis from purely coding to purely residual dimensions (see Methods). (f) Same as (e) for right trials. (g) Mean choice projection during the late delay period (215–235 ms) indicated by a gray rectangle in panel (e) as a function of the proportion of coding versus residual components in the perturbation. (h) Same as (g) for right trials.

We reasoned that since trial-by-trial variability in our network arises from fluctuations in input biases along both coding and residual dimensions, then it should be possible to identify the direction in input bias space that best separates correct and incorrect choices. We refer to this direction as the error axis: the axis in input bias space that most reliably predicts decision errors, which in principle could be different for left and right trials (Fig. 6a). To isolate this axis, we trained linear decoders to distinguish hit from miss trials separately for left and right choices, using trial-by-trial input biases extracted from the trained network (Fig. S6a, b; 91% and 93% cross-validated decoder accuracy, respectively). Importantly, the error axis is a vector in the input space (of dimension P), in contrast to the coding and residual dimensions, which lie in the neuronal activity space (of dimension N).

The error axes for left and right classifiers were highly correlated (Fig. 6b; Pearson correlation between decoder weights: r=−0.49), suggesting the presence of a shared component of the left and right error axes that drives decision errors across conditions. Analyzing the error axes loadings revealed contributions from both coding and residual dimensions (Fig. 6b). This suggest that trial-by-trial variability in residual dimensions may contribute to fluctuations in decision outcomes.

To test the causal role of trial-by-trial fluctuations along the error axes, we systematically varied the network’s input biases in those directions. These perturbations reliably shifted the choice-projected trajectories, converting left trials into right trials and vice versa (Fig. 6c,d; Fig. S6c-e). Perturbations along the error axes gradually shifted the choice projection toward the opposite choice (Fig. 6c), consistent with the gradual variations observed across left, right, and error trials (Fig. S6g,j). In contrast, the response projection exhibited a discrete boundary separating left from right choices, and perturbations along the error axis flipped trajectories across this boundary (Fig. 6d; Fig. S6h,k). Inputs along the error axes also induced continuous changes in the sample projection during the sample period in the direction of the opposite choice (Fig. S6c), although projections onto the sample dimension alone provided only weak discriminative power between hits and misses during this period (Fig. S6f,i).

To directly test whether residual dimensions causally drive decision errors, we systematically modified the composition of the input perturbation between coding and residual components while keeping its overall magnitude constant (Methods). Specifically, we fixed the total input bias magnitude that reliably produced choice reversals and continuously varied the relative contribution of coding and residual dimensions. Input biases confined to the coding subspace were insufficient to drive consistent errors in the network, whereas inputs containing an increasing proportion of residual components reliably induced erroneous choices in both left and right trials (Fig. 6e-h). These results demonstrate that input biases along residual subspace are necessary to shift the network toward incorrect trajectories, confirming their causal role in generating decision errors.

Together, these findings demonstrate that, in our trained networks, fluctuations in residual dimensions constitute a primary source of variability in choice that can causally bias single-trial decisions.

### Residual dimensions in thalamus shape cortical dynamics underlying decisions

Previous work has shown that the thalamocortical loop connecting ALM with the ventromedial (VM) and ventrolateral (VL) thalamic nuclei is causally involved in this decision-making task (Guo et al. 2017). We have shown in our ALM cortical model that residual dimensions play a causal role in shaping decisions. But what role do they play in communication across the thalamocortical loop?

One possibility is that choice-selective neurons in the motor thalamus influence ALM through selective, choice-specific interactions. In contrast, we find that even weakly selective thalamic populations can influence cortical decision dynamics if they project onto residual dimensions. This suggests that thalamic contributions to choice may arise from interactions within residual subspaces.

**Figure 7:**
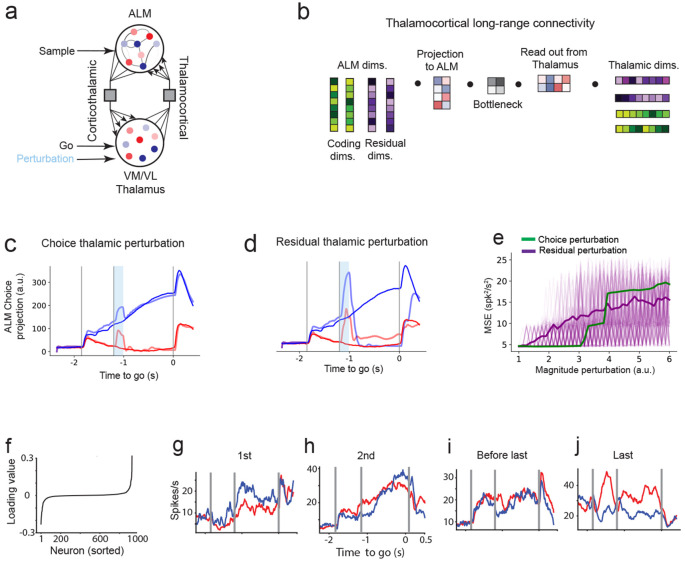
Thalamic residual dimensions shape cortical decision trajectories in a thalamocortical network model. (a) Schematic of the thalamocortical mesoscopic model, where ALM is modeled as a low-rank recurrent network (Fig. 1b) and thalamus as a non-recurrent population reciprocally connected via low-dimensional long-range connectivity. Stimulus was delivered through cortex, while the go cue was routed via thalamus. Perturbations are applied in thalamus. (b) Illustration of the low-dimensional thalamocortical long-range connectivity. (c–d) Choice projection following perturbations in thalamus along the choice dimension (c) and a residual dimension (d). Yellow bar indicates the perturbation period. (e) Mean squared error (MSE) between unperturbed and perturbed trajectories for 120 perturbations along residual (purple) and choice (green) dimensions at increasing magnitudes. Light traces show individual perturbations; solid lines indicate the average. (f) Sorted loadings of thalamic neurons onto the residual dimension shown in (d). (g–j) Trial-averaged activity of neurons with the highest (g–h) and lowest (i–j) loadings from (f) during correct left and right trials.

To examine whether residual dimensions in thalamus exert causal control over decision-making, we extended our ALM model into a thalamocortical circuit model (Fig. 7a, Methods). As before, the cortex was modeled as a low-rank recurrent network capturing both coding and residual dimensions (Fig. 1b). In line with anatomical evidence, we modeled the thalamus as a non-recurrent population of neurons that receives and sends long-range input to and from cortex. Thalamocortical and corticothalamic long-range connections were modeled as low-dimensional bottlenecks: they read out population activity from thalamus (or cortex) along specific lower-dimensional combinations of coding and residual dimensions, and project it into a lower-dimensional subspace of the connected area (Fig. 7b). This modeling approach builds on experimental and modeling work supporting that long-range projections transmit only a subset of the population activity dimensions, typically lower-dimensional than the full latent space, while the rest do not propagate across areas (Semedo et al. 2019; Barbosa et al. 2023; Pereira-Obilinovic et al. 2024; MacDowell et al. 2025). Finally, in line with experimental findings, the stimulus was delivered through cortex (Finkelstein et al. 2021), while the go cue was routed via thalamus (Inagaki et al. 2022).

The network was trained to simultaneously reproduce the trial-averaged activity of ALM and ventral thalamic neurons during correct trials (Fig. S7a, b). Consistent with experimental findings showing that the thalamocortical loop is necessary to sustain neural dynamics during the delay period (Guo et al. 2017), we found that disconnecting either the corticothalamic or thalamocortical projections led to a marked reduction on neural activity in the model (Fig. S7c).

Perturbations along the choice dimension in thalamus (Fig.7c, b), despite its strong correlation with behavior, had little effect on the final decision. In contrast, perturbing a subset of residual dimensions, which have minimal choice selectivity, consistently disrupt the choice projection (Fig. 7d, e). These effects were robust across hemispheres (Fig. S7d-f).

We examined how individual thalamic neurons contributed to the influential residual dimensions identified in our model. Similarly to the results in the trained models of ALM (Fig. S2q), neurons with strong loadings onto these dimensions did not exhibit high choice selectivity (Fig. 7f-j), indicating that the causal influence of these thalamic neurons was not aligned with choice tuning.

Together, these results demonstrate that, within the thalamocortical loop, residual dimensions in thalamus can causally influence decision dynamics. This supports a circuit mechanism in which broadly tuned, weakly selective thalamic populations drive the emergence of cortical selectivity (see Discussion)

## Discussion

Our analyses show that during a decision-making task, coding subspaces do not autonomously generate the observed dynamics. Although these dimensions account for a sizable fraction of the population variance, they depend on interactions with other, often weakly selective and low-variance residual dimensions. These residual dimensions lie outside task-coding subspaces and are typically dismissed as noise or irrelevant variability in dimensionality-reduction analyses. However, our results show that they actively drive the dynamics, and perturbing them can flip the choice outcome. The mechanism for this phenomenon is non-normal amplification of neural trajectories that occupy a relatively high number of latent dimensions (~10). Weak selectivity along the residual dimensions is dynamically amplified through non-normal interactions in the underlying network. As a result, even small perturbations along residual dimensions, which are dominated by neurons with low firing rates and weak task selectivity, can transiently grow and redirect trajectories within the coding subspace. This mechanism explains how seemingly untuned or weakly selective populations can exert strong effects on choice, as recently demonstrated by targeted optogenetic perturbations (Daie et al. 2023).

### A unified dynamical regime underlying ALM delay activity

Our fitted ALM models reveal a dynamical regime that unifies two dynamical mechanisms long considered substrates of short-term memory and decision-making. The network displays strongly non-normal delay dynamics (Goldman 2009; Ganguli et al. 2008; Orhan & Ma 2019; Stroud et al. 2024), yet these trajectories reliably collapse onto two choice-selective attractor states (Amit & Brunel 1997; Wang 2002; Wong & Wang 2006; Compte et al. 2000; Wimmer et al. 2015). Thus, our data-driven models unify several key observations about ALM dynamics: the strongly non-normal delay activity characterized in targeted perturbation experiments (Daie et al. 2023), the discrete attractor structure demonstrated in random-delay tasks (Inagaki et al. 2019; Oña-Jodar et al. 2024) and in distractor studies (Finkelstein et al. 2021), and the slowdown of neural activity observed in catch trials when the go cue is omitted.

These fitted dynamics also align closely with the normative theories for how recurrent networks solve delayed-response tasks (Orhan & Ma 2019; Stroud et al. 2023). These works shows that networks trained in delayed-response tasks with fixed go-cue timing—in contrast to paradigms with unpredictable delay durations—naturally adopt non-normal dynamics to optimally load sensory information (Stroud et al. 2023): stimulus inputs initially drive activity along amplifying non-normal dimensions, and only later do trajectories collapse onto fixed-point attractors during the delay. Our ALM models exhibit precisely this organization in their dynamics. Together, our results reveal a unified dynamical motif in ALM: non-normal transients that funnel activity into choice-selective attractors, with amplification and stability that may be flexibly modulated depending on task demands.

### Residual dimensions shape decision trajectories

More generally, our findings suggest that classical decision-making models, which often reduce population activity to two latent dimensions aligned with stimulus or choice encoding (Wang 2002; Wong & Wang 2006), may not fully capture the structure of neural dynamics in some decision-making tasks. A similar reduction underlies several data analyses (Mante et al. 2013; Kobak et al. 2016; Inagaki et al. 2019), as well as recent mechanistic, data-driven models that focus on modeling dynamics restricted to low-dimensional, task-aligned subspaces (Langdon & Engel 2025; Luo et al. 2025). While these approaches improve interpretability and provide conceptually simple descriptions of task-related activity, they may overlook essential aspects of the population dynamics.

The residual dimensions identified here indicate that neural circuits operate within intermediate-dimensional dynamical subspaces, where additional degrees of freedom interact with coding dimensions to shape decisions. Partial observation can cause data-driven models to spuriously infer line-attractor structure even when the underlying circuit is non-normal (Qian et al. 2024). In our case, the recovered dynamics remain strongly non-normal, suggesting that our model operates at a dimensionality that is in the similar range as that of the ALM delay-period dynamics. This view aligns with recent findings in the monkey motor cortex during reaching, where intermediate-dimensional (15-25) non-normal dynamics drive transient activity (O’Shea et al. 2022). Our findings also relate to studies in motor cortex, where preparatory activity unfolds in movement-null subspaces that do not directly drive outputs but modulate execution (Kaufman et al. 2014; Elsayed et al. 2016; Khilkevich et al. 2024). Similarly, we show that residual dimensions can causally shape decision-related dynamics without aligning with canonical choice subspaces.

Importantly, we show that in the delayed-response task, while the choice dimension remains relatively stable across the delay period (Li et al. 2016; Inagaki et al. 2018; Daie et al. 2023), neural activity along the choice dimension is not self-sustaining but instead driven by dynamics unfolding along both residual and coding dimensions. Choice-selective neurons therefore reflect the upcoming decision but do not control it. These results challenge the conventional view that low-dimensional coding subspaces are sufficient to explain neural computations and reveal that dimensions previously considered task-irrelevant can play a causal role in shaping behavior.

### Trial-to-trial variability as low-dimensional modulation of the global dynamical landscape

In contrast to previous data-driven models that treat trial-by-trial variability as time-varying independent and identically distributed noise (Sourmpis et al. 2023; Pals et al. 2024), we model variability as a non-stationary, low-dimensional modulation of the population dynamics that remains constant within each trial. By representing each trial as a small magnitude, constant input bias acting on both coding and residual dimensions, our framework captures how trial-specific fluctuations perturb the shared low-dimensional dynamics of the network. These perturbations reshape the network’s activity space, effectively reshaping neural trajectories across trials and changing the velocity of the non-normal dynamics, which can be selectively modulated by constant inputs, as recently demonstrated in theoretical models (Gillett & Brunel 2024). This modeling approach is particularly relevant in our task, where contingencies differ but the sensory stimulus within a given contingency is held constant across trials; unlike perceptual decision-making paradigms with trial-varying sensory evidence (Steinemann et al. 2024; Luo et al. 2025), variability here is more likely to reflect fluctuations in internal states (e.g., engagement or satiety) than changes in sensory input. Remarkably, we found that fluctuations along residual dimensions—rather than coding dimensions—were the primary drivers of decision errors, indicating that weakly selective neuronal populations within residual dimensions are causally responsible for fluctuations in single-trial decisions.

### Residual thalamic dimensions drive preparatory dynamics in cortex

Thalamocortical interactions are critical for maintaining preparatory activity in this task (Guo et al. 2017; Yang et al. 2022). Photoinhibition of motor thalamus causes a near-complete collapse of ALM activity (Guo et al. 2017), demonstrating its causal role in sustaining cortical dynamics. We considered two possible modes of thalamocortical communication that could generate choice selectivity. The first involves labeled-line projections where choice-selective thalamic neurons connect to matching selective populations in ALM, producing selective delay activity through reverberations within the loop. In this case, perturbations of choice-selective neurons in thalamus would be expected to strongly disrupt selectivity in ALM. The second involves residual projections where weakly selective thalamic neurons, dominated by high loadings in residual dimensions, drive ALM preparatory activity through recurrent thalamocortical interactions. In this case, perturbations along residual dimensions in thalamus would be expected to disrupt selectivity in ALM. Our perturbations in the thalamocortical model show that choice selectivity in the thalamocortical loop arises from residual projections that mediate communication between thalamus and ALM. These results suggest that weakly selective thalamic activity, potentially reflecting inputs from basal ganglia (Wang et al. 2021), may play a key role in controlling thalamocortical dynamics that generate ALM preparatory activity.

### Residual dimensions as control knobs for decision dynamics

From a normative perspective, our findings suggest that neural circuits may use residual dimensions as control knobs to shape decision trajectories. These dimensions could enable fine modulation of specific aspects of the decision process, such as its speed, amplitude, or stability. This view supports a broader reconceptualization of low-variance, weakly task-encoding components of neural activity not as noise, but as organized fluctuations in population activity that reflect flexible neural computations.

## Methods

### Experimental data and preprocessing

#### Neural recordings

We analyzed and trained our models on extracellular recordings from mice performing a delayed response decision-making task (Chen et al. 2024). Recordings were obtained from the anterior lateral motor cortex (ALM) and the ventromedial (VM) and ventrolateral (VL) thalamic nuclei, which are causally involved in this task (Guo et al. 2017). Neural activity was acquired across 176 behavioral sessions from 28 mice, yielding a total of 7,884 cortical neurons (3,640 left hemisphere, 4,244 right hemisphere) and 1,932 thalamic neurons (1,004 left hemisphere, 928 right hemisphere). For the single-trial fits, we used a session with 102 simultaneously recorded ALM neurons in the right hemisphere. Preprocessing steps followed standard filtering and spike sorting pipelines and full data provenance is reported in (Chen et al. 2024). Spikes were then smoothed using an exponential filter with a timescale of 50ms.

#### Identification of task-coding dimensions

To identify the coding subspace, we applied linear discriminant analysis (LDA) to condition-averaged neural trajectories from correct left and right trials (Li et al. 2016; Inagaki et al. 2019; Finkelstein et al. 2021; Chen et al. 2024). This analysis was performed separately for each hemisphere using pseudopopulations constructed by pooling neurons across sessions, resulting in 3,640 neurons for the left ALM and 4,244 neurons for the right ALM. Each trajectory was embedded in the full N-dimensional activity space of the corresponding pseudopopulation.

We extracted the dimensions that best separated left and right trajectories during three key behavioral epochs: (1) the sample dimension ${\vec{v}}_{\text{sample}}$, computed from the first 600 ms of the sample period; (2) the choice dimension ${\vec{v}}_{\text{choice}}$, computed from the 600 ms preceding the Go cue; and (3) the response dimension ${\vec{v}}_{\text{response}}$, computed from the 350 ms following the Go cue. The resulting vectors ${\vec{v}}_{\text{sample}}$, ${\vec{v}}_{\text{choice}}$, ${\vec{v}}_{\text{response}}$ were orthogonalized using the Gram–Schmidt procedure (Golub & Van Loan 2013) to ensure they formed an orthonormal basis for the coding subspace.

These dimensions were selected to reflect the core computational demands of the task: sensory integration (sample), choice maintenance (choice), and motor execution (response). Together, ${\vec{v}}_{\text{sample}}$, ${\vec{v}}_{\text{choice}}$, and ${\vec{v}}_{\text{response}}$ span a coding subspace that captures differences in neural dynamics across conditions at different epochs of the trial. We explicitly incorporated these coding dimensions into the network model through alignment constraints during training (see Training procedure on condition-averaged data).

### Network models

#### ALM model

We modeled ALM as a recurrent rate-based neural network governed by standard rate equations (Grossberg 1969; Hopfield 1982; Miller & Fumarola 2012):

(3) $$\tau \frac{dr_{i}}{dt}=-r_{i}+\phi_{i}\left(\sum_{j=1}^{N}J_{ij}r_{j}+I_{i}(t)\right),$$

where τ=40 ms is the neuronal integration time constant. The activation function $\phi_{i}(x)$ is given by

(4) $$\phi_{i}(x)=\frac{r_{i}^{\max}}{2}\left(\tanh(x)+1\right),$$

with $r_{i}^{\max}$ denoting the maximum firing rate of neuron i, treated as a trainable parameter.

The recurrent connectivity matrix $J_{ij}$ is constrained to be low-rank and takes the form:

(5) $$J_{ij}=\sum_{l=1}^{P}\sum_{l^{\prime }=1}^{P}A_{ll^{\prime }}u_{i}^{l}u_{j}^{l^{\prime }},$$

where the vectors ${\left\{{\vec{u}}^{l}\right\}}_{l=1}^{P}$ define the connectivity structure. We define the matrix U as the matrix whose columns are the coding and residual dimensions, such that $U=[{\vec{u}}_{1},{\vec{u}}_{2},\ldots ,{\vec{u}}_{P}]$. The first C vectors span the task-relevant coding subspace, while the remaining R=P−C vectors span the residual subspace. All vectors ${\vec{u}}^{l}$ are optimized to remain orthonormal during training (see below). This low-rank structure (Mastrogiuseppe & Ostojic 2018) has been widely adopted in models of associative memory (Hopfield 1982), sequence generation (Sompolinsky & Kanter 1986; Kleinfeld 1986; Gillett et al. 2020), and manifold-based attractor dynamics (Ben-Yishai et al. 1995; Darshan & Rivkind 2022).

The external input is given by:

(6) $$\vec{I}(t)={\vec{I}}_{\text{task}}(t)+{\vec{I}}_{\text{licks}}(t)$$

To match the task structure used in the experiments, the external input ${\vec{I}}_{\text{task}}(t)$ was defined as:

(7) I→task(t)={U⋅I→(sample),t∈[−1.85s,−1.7s)∪[−1.6s,−1.45s)∪[−1.35s,−1.2s)U⋅I→Go,t∈[0,0.1s)0,otherwise}

where time zero is the time of the go cue and with sample-dependent input defined as:

(8) I→(sample)={I→low,Low toneI→high,High tone0,otherwise.}

The P-dimensional input parameters ${\vec{I}}_{\text{low}}$, ${\vec{I}}_{\text{high}}$, and ${\vec{I}}_{\text{Go}}$ were optimized during training.

Lastly, we included a proprioceptive-like input representing licking activity, which was modeled as a time-varying input proportional to the average lick rate and differed between left and right trials:

(9) I→lick(t)={0,t≤0I→licks⋅Ratelick(t),t>0}

where the amplitude vector ${\vec{I}}_{\text{licks}}$ was determined by trial type:

(10) I→licks={I→lick-left,Left choiceI→lick-right,Right choice.}

The parameters ${\vec{I}}_{\text{lick-left}}$ and ${\vec{I}}_{\text{lick-right}}$ were optimized during training. As indicated by Eq. (9), lick-related inputs only influence the network after the go cue, consistent with the absence of anticipatory licking in behavior. This input captures the strong sensory feedback that licking exerts on ALM, while acknowledging that the motor act of licking originates from downstream structures (Kaku et al. 2025). Thus, we interpret this signal as a proprioceptive input reflecting ongoing motor output. The rationale for including measured licking activity is to prevent the ALM model from having to generate lick-evoked responses that originate outside ALM. However, because the model was trained only up to 350 ms post-go cue, when few licks occur, this input had minimal impact on the the network dynamics.

By projecting the network dynamics in Eq. (3) onto the coding and residual subspaces, we obtained a reduced description of population activity in terms of latent variables ${\left\{m_{s}\right\}}_{s=1}^{P}$, corresponding to the projections of firing rates onto the low-rank basis (Eq. (5)). The resulting latent dynamics evolve as:

(11) $$\tau \frac{dm_{s}}{dt}=-m_{s}+\sum_{j=1}^{N}u_{j}^{s}\phi_{j}\left(\sum_{l=1}^{P}\sum_{l^{\prime }=1}^{P}u_{j}^{l}A_{ll^{\prime }}m_{l^{\prime }}+I_{j}(t)\right),$$

In our implementation, the number of trainable parameters includes N×P for the low-rank basis vectors $u_{j}^{l}$, P×3 task input vectors, N×2 licking input vectors, N maximum firing rates, and $P^{2}$ parameters for the interaction matrix A.

For low-dimensional neural dynamics, where P≪N, this low-rank parametrization dramatically reduces the number of trainable parameters to 𝒪(N) compared to full-rank models, which require $𝒪(N^{2})$ parameters.

### Mathematical analysis of the interaction matrix A

#### Henrici’s departure from normality

To quantify the degree of non-normality in the dynamics matrix A, we used the normalized Henrici’s departure from normality (Asllani et al. 2018). This index measures how much a matrix deviates from being normal, that is, from satisfying ${\mathrm{AA}}^{T}=A^{T}A$, which is equivalent to being unitarily diagonalizable with orthogonal eigenvectors (Golub & Van Loan 2013). The normalized form of Henrici’s measure ranges between 0 and 1.

For a square matrix $A\in R^{P\times P}$, it is defined as

(12) $$d_{F}(A)=\frac{1}{{\left\|A\right\|}_{F}}\sqrt{{\left\|A\right\|}_{F}^{2}-\sum_{i=1}^{P}{|\lambda_{i}|}^{2}},$$

where ${\left\|A\right\|}_{F}=\sqrt{\sum_{i,j}{|A_{ij}|}^{2}}$ is the Frobenius norm and ${\left\{\lambda_{i}\right\}}_{i=1}^{n}$ are the eigenvalues of A.

By construction, $d_{F}(A)=0$ if and only if A is normal, in which case the eigenvalues fully capture the matrix’s structure. Larger values indicate stronger departures from normality, with $d_{F}(A)=1$ obtained when all eigenvalues vanish and A is nilpotent (e.g. consisting of Jordan blocks with eigenvalue zero). In this extreme case, the spectrum provides no information about the matrix’s behavior, and all the linear dynamics arise from non-orthogonal interactions between eigenvectors (Ganguli et al. 2008; Goldman 2009). While this interpretation is exact for linear systems, in nonlinear networks non-normal interactions can similarly drive strong transient activity (Gillett et al. 2020).

#### Pseudospectra analysis

To quantify the non-normal structure of the linear interactions induced by the interaction matrix A, we computed the pseudospectrum. The pseudospectrum characterizes how sensitive a linear system induced by the matrix A is to small perturbations and is defined as the set of complex values z for which the resolvent norm

(13) $${\left\|{\left(zI-A\right)}^{-1}\right\|}_{2}$$

is larger than a threshold (Trefethen & Embree 2020). This norm was computed as the largest singular value of ${\left(zI-A\right)}^{-1}$ over a grid of complex values z=x+iy surrounding the eigenvalue spectrum of A. Contours of $\log_{10}{\left\|{\left(zI-A\right)}^{-1}\right\|}_{2}$ visualize regions where perturbations to coding and residual dimensions are most strongly amplified.

For a normal matrix, whose eigenvectors are orthogonal, the resolvent norm depends solely on the distance between z and the eigenvalues,

$${\left\|{\left(zI-A\right)}^{-1}\right\|}_{2}=1∕\underset{i}{\text{min}}|z-\lambda_{i}|.$$

where ${\left\{\lambda_{l}\right\}}_{l=1}^{P}$ correspond to the eigenvalues of A. For a non-normal A, where eigenvectors are non-orthogonal, the resolvent norm can become large even far from the spectrum (eigenvalues), revealing directions in latent space where transient amplification occurs. In our model in Eq. (3), the resolvent captures, up to a gain modulation introduced by the network’s nonlinearity, the linearized response of the dynamics to small perturbations in neural activity (see Eq. (18)).

To relate pseudospectral amplification to coding and residual subspaces, we quantified the alignment between the most amplifiable directions and the residual subspace. For each complex z, we computed the top right singular vector ${\vec{v}}_{1}(z)$ of ${\left(zI-A\right)}^{-1}$, corresponding to the input direction that produces maximal amplification. The overlap between ${\vec{v}}_{1}(z)$ and the residual subspace, spanned by the P−C dimensional orthonormal basis R, was quantified as

(14) $$\text{Alignment}(z)={\left\|R{\vec{v}}_{1}(z)\right\|}^{2},$$

ranging from 0 (fully aligned to the coding dimensions) to 1 (fully aligned to the residual dimensions). This alignment map in Fig. 3l is used to identify which subspaces support maximal transient amplification.

In our fitted models, regions of strongest pseudospectral amplification, corresponding to areas in the complex plane near eigenvalues with positive real parts and with strong shared non-normality among eigenvectors, indicative of non-normal transient amplification, consistently aligned with residual dimensions (Fig. 3l and also b). This analysis shows that transient amplification predominantly acts along these residual dimensions.

#### Schur decomposition and analysis of interaction modes

To characterize the structure of the interaction matrix A, we computed its Schur decomposition,

(15) $$A={\mathrm{QTQ}}^{T},$$

where Q is an orthogonal matrix whose columns form an orthonormal basis of activity modes, and T is a quasi–lower-triangular matrix whose diagonal entries correspond to the eigenvalues of A. In the real Schur decomposition, complex-conjugate eigenvalues appear as 2 × 2 real blocks on the diagonal of T, where the off-diagonal element encodes the imaginary part of the eigenvalues and represents the coupling between the corresponding Schur modes. The strictly lower off-diagonal elements of T quantify the hidden feedforward structure in the interaction matrix A and reflect its degree of non-normality (Hennequin et al. 2012). These terms capture how activity along one Schur dimension can transiently drive or amplify activity in another.

We obtained the Schur-projected activity by projecting the latent variables m(t) (see Eq. (2)) onto the Schur basis,

(16) $$s_{l}(t)=\sum_{l^{\prime }=1}^{P}Q_{ll^{\prime }}m_{l^{\prime }}(t),$$

where each component $s_{l}(t)$ represents the temporal evolution of the l-th Schur mode.

The Schur decomposition is not unique. Within invariant subspaces corresponding to degenerate or complex-conjugate eigenvalues, the columns of Q can be freely rotated without changing the decomposition (Golub & Van Loan 2013). In addition, the Schur form allows reordering of the modes, since permuting the diagonal blocks of T and the corresponding columns of Q preserves $A={\mathrm{QTQ}}^{T}$. We used this freedom to order the Schur dimensions such that eigenvalues with negative real parts appear first in T and those with positive real parts last, ensuring consistent grouping of Schur dimensions across networks.

#### Network perturbations to coding and residual dimensions

To assess the causal contribution of individual network dimensions, we applied brief input perturbations aligned with either coding or residual dimensions during the delay period (Fig. 2). Perturbations along the coding and residual dimensions were of the form $I_{\text{pert}}=g{\vec{u}}^{l}$, where g is the perturbation magnitude and ${\vec{u}}^{l}$ is the l-th network dimension. Random perturbations were generated within the subspace orthogonal to the coding and residual dimensions by projecting random vectors onto the column space of $U^{⊥}$, whose columns, obtained via QR decomposition of U, form an orthonormal basis of its complement.

In Fig. 2g, each perturbation lasted 100 ms and was applied at one of 12 evenly spaced time points during the delay period. We tested networks with latent dimensionality ranging from 9 to 16, applying perturbations across all learned dimensions. Each perturbation condition was run on 40 trials, with magnitudes ranging from g=−15 to g=15. The Go cue was withheld on both perturbed and unperturbed trials, allowing the network dynamics to evolve toward the corresponding condition-specific fixed point. Simulations ran for 0.9 s following the nominal Go cue time, which in most cases was sufficient for the network to closely approach to the the condition-selective attractor states.

To quantify the effect of perturbations, we computed the *rate of choice flips*, defined as the fraction of trials in which the unperturbed activity, when projected on the choice dimension, differed from the choice-projected perturbed activity. Let $x_{L}$ and $x_{R}$ denote the endpoints of the unperturbed dynamics for left and right trials, respectively. That is, the network output at the end of the delay period. Let ${\hat{x}}_{L}$ and ${\hat{x}}_{R}$ denote the corresponding outputs for perturbed trials. We defined a symmetric threshold margin:

(17) $$\epsilon =0.25\cdot (x_{R}-x_{L})$$

A flip toward right was counted if $|{\hat{x}}_{L}-x_{R}|<\epsilon$, and a flip toward left if $|{\hat{x}}_{R}-x_{L}|<\epsilon$. Flip rates were averaged across perturbation trials for each dimension, time point, and magnitude. For Fig. 2g and Fig. 3h, we computed the rate of choice flips for 100ms perturbations applied at distinct time windows (during the delay for Fig. 2h; during the sample and delay periods for Fig.3g) across network dimensions. For each dimension, the perturbation amplitude was varied from −15 to 15. In Fig. 2h, perturbations were aligned with the choice and residual dimensions; in Fig. 3h, they aligned with the Schur dimensions (see Mathematical analysis of the interaction matrix).

#### Network perturbations targeting choice- and residual-selective neurons

Current experimentally feasible manipulations act on subsets of neurons rather than specific dimensions in neuronal state spaces (Daie et al. 2021). To mirror this constraint, we targeted neurons most aligned with either the choice or residual subspaces, instead of perturbing the macroscopic dimensions themselves.

Using the decoder matrix U (Eq. (5)), we computed per-neuron selectivity with respect to the choice and residual dimensions by sorting the loadings of these dimensions. For a given group size K∈{1,…,30}, we selected the top-K neurons by the chosen ranking (“choice-selective” or “residual-selective”).

Perturbations were implemented as an external input ${\vec{I}}_{\text{pert}}$ delivered during the first 100 ms of the delay period (see Fig. 2j-l). For a target class (choice or residual), group size K, and magnitude α∈{10,20,30}, each neuron in the selected set received input of amplitude $\alpha ∕\sqrt{K}$. Thus, ${\vec{I}}_{\text{pert}}$ is K-hot with constant norm across $K:{\left\|{\vec{I}}_{\text{pert}}\right\|}_{2}=\alpha$.

To quantify the effect (Fig. 2j), we computed the absolute difference between the left- and right-trial trajectories projected onto the choice axis, averaged over the 100 ms preceding the Go cue, for perturbations to choice-selective and residual-selective subsets.

#### Stability analysis of fixed-point attractors

To assess the stability of condition-specific fixed points reached during the delay period, we analyzed the linearized dynamics of the trained ALM network models using the Jacobian evaluated at the final state of the trial-averaged trajectories.

We evaluated models trained on right hemisphere ALM activity, with latent dimensionalities ranging from P=9 to P=16, including multiple random initializations per dimensionality. For each model, we simulated the network response to trial-averaged inputs corresponding to left and right conditions, withholding the Go cue, and allowed the network to evolve toward a fixed point. To ensure convergence, we extracted the final network state at time 1.05s after the Go cue.

The Jacobian was computed by linearizing the dynamics in Eq. (3) around the final state. The linearized system governing small perturbations $\delta \vec{r}$ near the fixed point takes the form:

(18) $$\tau \frac{d\;\delta \vec{r}}{dt}=-\delta \vec{r}+D\cdot J\cdot \delta \vec{r},$$

where D is a diagonal matrix of derivatives of the activation function $\phi_{i}$ (Eq. (4)), and J is the network connectivity matrix:

(19) $$J={\mathrm{UAU}}^{T}.$$

The gain-modulated activation derivative for each neuron is given by:

(20) Dij={rimax2(1−tanh2(hi)),i=j,0,i≠j.}

where $h_{i}=\sum_{j}J_{ij}r_{j}+I_{i}(t)$ is the input current to neuron i at the fixed point.

The Jacobian matrix, M=−I+DJ, was constructed using Eqs. (19,20) and used to compute the eigenvalues separately for left and right conditions. The eigenvalue with the largest real part determined the local stability of the fixed point (Guckenheimer & Holmes 2013): negative real parts indicate stability, while values near zero or positive indicate marginal or unstable dynamics.

This analysis was repeated across all trained models, yielding a distribution of maximal eigenvalues as a function of latent dimensionality and random seed (Fig. S4a, which corresponds to 48 trained networks), quantifying the stability of task-specific attractor states in our low-rank models of ALM.

#### Training procedure on condition-averaged data

We trained the network model described in Eqs. (3-11) to reproduce trial-averaged neural dynamics recorded in the ALM during a memory-guided decision-making task only for hit (correct) trials. The training objective minimized a weighted sum of three loss terms:

1. **Reconstruction loss**. This term minimized the mean squared error between the model’s predicted firing rates, ${\vec{r}}_{L}(t)$ and ${\vec{r}}_{R}(t)$, and the condition-averaged neural data, ${\vec{\bar{y}}}_{L}(t)$ and ${\vec{\bar{y}}}_{R}(t)$, for left and right choice trials:
(21) $${\text{Loss}}_{\text{recon}}={\left\|{\vec{r}}_{L}(t)-{\vec{\bar{y}}}_{L}(t)\right\|}_{2}^{2}+{\left\|{\vec{r}}_{R}(t)-{\vec{\bar{y}}}_{R}(t)\right\|}_{2}^{2}.$$
2. **Coding alignment loss**. The model was trained to align its coding dimensions ${\left\{{\vec{u}}^{l}\right\}}_{l=1}^{C}$ to match the axes extracted from the neural recordings that optimally encode sample, choice, and response using LDA ${\vec{v}}_{\text{sample}}$, ${\vec{v}}_{\text{choice}}$, ${\vec{v}}_{\text{response}}$. For this we introduced a cosine similarity loss:
(22) $${\text{Loss}}_{\text{align}}=4-\cos({\vec{u}}^{1},{\vec{v}}_{\text{sample}})-2\cdot \cos({\vec{u}}^{2},{\vec{v}}_{\text{choice}})-\cos({\vec{u}}^{3},{\vec{v}}_{\text{response}}).$$
  The cosine similarity term for the choice axis was weighted more heavily (multiplied by 2) to prioritize alignment with the choice dimension, which serves as the network’s primary readout for upcoming decisions.
3. **Orthogonality loss.** To enforce orthogonality among the low-rank connectivity dimensions, we penalized deviations of the decoder matrix U from an orthonormal basis:
(23) $${\text{Loss}}_{\text{orth}}={\left\|I-U^{T}U\right\|}_{2}^{2}.$$
  The total loss combined these terms:
(24) $$\text{Total Loss}=\lambda_{\text{recon}}\cdot {\text{Loss}}_{\text{recon}}+\lambda_{\text{align}}\cdot {\text{Loss}}_{\text{align}}+\lambda_{\text{orth}}\cdot {\text{Loss}}_{\text{orth}}.$$

Loss weights were set to $\lambda_{\text{recon}}=0.7$, $\lambda_{\text{align}}=0.5$, and $\lambda_{\text{orth}}=500$. These values were chosen based on empirical performance across fits.

Training was performed using the Adam optimizer with a learning rate of 5 × 10^−4^ for up to 400,000 iterations. Gradients were clipped to improve training stability. Separate models were trained for each hemisphere using custom scripts in PyTorch.

All models were implemented in PyTorch 2.4.0 with CUDA 12.4.0 and trained on an NVIDIA T4 GPU. Each model converged in under approximately 36 hours. We trained multiple models per hemisphere across a range of latent dimensionalities (P=3−20) and at least five random seeds per setting.

#### Cross-validation for networks trained on trial-averaged neural recordings

To assess generalization, we performed cross-validation by repeatedly splitting the dataset at the trial level. For each fold, 80% of the trials were randomly sampled to compute condition-averaged neural activity and train the network. The remaining 20% were held out to evaluate model performance.

This procedure was repeated across five independent random splits using different seeds. For each fold, we computed the mean squared error (MSE) between the network’s predicted firing rates and the trial-averaged activity in the held-out test set. We also computed the cosine similarity between the coding dimensions extracted from the network and those derived from the data using LDA, both computed on the training set. All reported metrics reflect the average and standard error across folds (Figs. 1 and S1).

This procedure was repeated across a range of latent dimensionalities and separately for each hemisphere (Fig S1) and averaged over hemispheres (Fig 1). The results were smoothed using a five-point moving average.

#### Model of single-trial variability

To capture trial-by-trial variability, we modeled each trial as receiving a low-dimensional, trial-specific input current. Specifically, on trial k, the input to neuron i was defined as:

(25) $$I_{i}^{\left(k\right)}=\sum_{l=1}^{P}u_{i}^{l}T_{lk},$$

where $T_{lk}$ is a trial-specific matrix of coefficients, and ${\left\{{\vec{u}}^{l}\right\}}_{l=1}^{P}$ are the low-rank basis vectors spanning the coding and residual subspaces (Eq. (5)). These inputs define a constant offset along the low-dimensional subspace for each trial, shifting the network’s operating point without modifying its connectivity.

The rate dynamics $r^{\left(k\right)}$ for neuron i on trial k were then governed by:

(26) $$\tau \frac{dr_{i}^{\left(k\right)}}{dt}=-r_{i}^{\left(k\right)}+\phi_{i}\left(\sum_{j=1}^{N}J_{ij}r_{j}^{\left(k\right)}+I_{i}(t)+I_{i}^{\left(k\right)}\right).$$

The inputs $I_{i}^{\left(k\right)}$ remained fixed throughout each trial but varied across trials. This formulation introduces variability across trials without modifying the recurrent connectivity.

These inputs can be interpreted as introducing small, trial-specific deformations in the vector field governing the dynamics in Eq. (3). Because the variability is restricted to the coding and residual subspaces, it remains interpretable and aligned with the dominant dimensions that shape population activity.

This approach was motivated by the non-stationary fluctuations observed in neural recordings (Fig. 5g-j), which likely reflect changes in internal states such as arousal, engagement, or satiety, which are factors known to modulate cortical activity (Poulet & Petersen 2008; Matteucci et al. 2022; Steinmetz et al. 2019; Allen et al. 2019). Unlike additive independent and identically distributed noise models, this formulation captures trial-to-trial variability as a low-dimensional, structured modulation of the network dynamics.

#### Training procedure on single-trial data

We trained the single-trial model described in Eq. (26) to capture structured, low-dimensional variability across trials. The single-trial neural data were represented as a tensor $y_{kti}$, where k indexes trials, t time points, and i neurons. Training was performed by jointly optimizing the parameters of the recurrent network (Eqs. (3-11)) and the trial-specific input currents $I_{i}^{\left(k\right)}$ (Eq. (25)) to reproduce summary statistics of this tensor. These trial-specific inputs perturb the network along low-dimensional directions within the coding and residual subspaces (Eq. 5), enabling the model to capture trial-to-trial variability in an interpretable manner aligned with the network’s low-dimensional structure.

In addition to the low-dimensional, constant trial-specific input currents, the model also received the condition-averaged task inputs described in Eqs.(6-10), as well as convolved single-trial lick signals. These lick inputs were weighted separately for left and right responses using the trained input weights defined in Eqs.(9, 10). They were introduced to match lick-related activity likely originating from subcortical structures (Kaku et al. 2025) and influenced the network dynamics only after the Go cue, since animals did not lick during the sample or delay periods. As in the trial-averaged network, including measured licking activity prevents the ALM model from having to generate lick-evoked responses internally; omitting these inputs would otherwise force the model to produce activity that originates outside ALM.

To prevent the model from learning a direct mapping from the trial-specific input current $I_{i}^{\left(k\right)}$ to the responses, we jittered the trial start time at each training epoch. On each trial k, inputs began at an offset independent and identically uniformly distributed across trials $t_{\text{start}}^{\left(k\right)}\overset{\text{i.i.d.}}{∼}𝒰(-4.35,-2.35)\;s$ relative to the go cue. Training networks with a fixed start time, they typically exploited input biases to produce transient activity that fits the data without integrating stimulus information to generate delay activity. Jittering the start time during training prevents the network from finding this solution and forces it to integrate stimulus information over time rather than relying on $I_{i}^{\left(k\right)}$ alone. After training, removing the stimulus inputs caused the trained networks to fail to reproduce the variable ramping of the choice latent characteristic of single-trial neural activity, indicating that integration of sample information is required to generate preparatory activity (Fig. S5).

Rather than fitting individual spike trains, we trained the model to reproduce multiple contractions of the neural activity tensor $y_{kti}$. This approach leverages the strengths of rate-based modeling while capturing critical statistical features of trial-to-trial variability inherent in spiking data. Our primary goal was to uncover low-dimensional neural structures that generalize across trials. Direct fitting to individual spike trains risks overfitting to trial-specific noise, limiting the model’s generalization. In contrast, training the model using summary statistics (tensor contractions) emphasizes shared structure across trials, promoting robust learning of stable latent dynamics and interpretable low-dimensional variability (Pellegrino et al. 2024) (Fig. 5). Additionally, we explicitly included contractions aimed at capturing variability within the coding dimensions. This allowed us to investigate mechanisms underlying trial-to-trial variability specifically within task-relevant dimensions, such as the choice axis, providing insights particularly relevant to the analysis of error trials. The following losses were minimized:

1. **Trial-averaged loss.** Matches the model firing rates to the condition-averaged neural activity across trials, computed separately for left and right choice conditions as in Eq. (21).
2. **Epoch-averaged loss.** Matches average firing rates across trials and neurons within task-defined temporal epochs:
(27) $${\text{Loss}}_{\text{epoch}}=\sum_{e}\sum_{k,i}{\left({\bar{y}}_{ki}^{\left(e\right)}-{\bar{r}}_{ki}^{\left(e\right)}\right)}^{2},$$
  where ${\bar{y}}_{ki}^{\left(e\right)}=\frac{1}{|t\in e|}\sum_{t\in e}y_{kti}$ and ${\bar{r}}_{ki}^{\left(e\right)}=\frac{1}{|t\in e|}\sum_{t\in e}r_{kti}$ are the trial- and epoch-averaged firing rates for neuron i on trial k during epoch e∈{sample,delay,response}.
3. **Projection loss.** Matches the model’s projections onto the coding dimensions ${\left\{{\vec{u}}^{l}\right\}}_{l=1}^{C}$ to the single-trial neural activity projected onto LDA-computed coding dimensions:
(28) $${\text{Loss}}_{\text{proj}}=\sum_{k,t}\sum_{l\in \{\text{sample},\text{choice},\text{response}\}}{\left({\vec{m}}_{kt}^{l}-{\vec{y}}_{kt}^{T}{\vec{v}}_{l}\right)}^{2},$$
  where ${\vec{y}}_{kt}$ is the observed population activity on trial k at time t, and ${\vec{v}}_{l}$ is the LDA-derived coding axis for dimension l∈{sample,choice,response}. The model projections ${\vec{m}}_{kt}^{l}$ are computed as the dot product between the model’s firing rates and the corresponding coding direction: ${\vec{m}}_{kt}^{l}={\vec{r}}_{kt}^{T}{\vec{u}}^{l}$. The coding dimensions $\left\{{\vec{u}}^{l}\right\}$ and the LDA vectors $\left\{{\vec{v}}_{l}\right\}$ are encouraged to be aligned via the coding alignment loss (Eq. (22)) but are not necessarily identical. This loss encourages the model to reproduce trial-by-trial variability along coding dimensions and further reinforces alignment between the model’s learned coding subspace and the LDA-derived axes.
4. **Coding alignment loss.** See Eq. (22).
5. **Orthogonality loss.** See Eq. (23).
6. **Input regularization.** This term penalized the overall magnitude of the trial-specific inputs to constrain their influence on the dynamics:
(29) $${\text{Loss}}_{\text{reg}}={\left\|T\right\|}_{2}^{2},$$
  where T is a matrix with one column per trial and one row per low-dimensional input direction (Eq. (25)). This constraint encourages the model to account for trial-by-trial variability through modest perturbations, ensuring that most structure arises from the low-dimensional dynamics.
  The total loss was a weighted sum of all six components:
(30) $$\text{Total Loss}=c_{\text{recon}}\cdot {\text{Loss}}_{\text{recon}}+c_{\text{epoch}}\cdot {\text{Loss}}_{\text{epoch}}+c_{\text{proj}}\cdot {\text{Loss}}_{\text{proj}}+c_{\text{align}}\cdot {\text{Loss}}_{\text{align}}+c_{\text{orth}}\cdot {\text{Loss}}_{\text{orth}}+c_{\text{reg}}\cdot {\text{Loss}}_{\text{reg}}.$$

Loss weights were selected empirically (manual tuning to promote stable optimization and a consistent decrease in the loss). We used $c_{\text{recon}}=0.65$, $c_{\text{epoch}}=0.1666$, $c_{\text{proj}}=0.02$, $c_{\text{align}}=0.5$, $c_{\text{orth}}=500$, and $c_{\text{reg}}=0.1$. To stabilize training, full loss terms were activated only after the trial-averaged loss fell below a threshold.

Training used the Adam optimizer with learning rate 5 × 10^−4^ and gradient clipping. All models were implemented in PyTorch and trained on NVIDIA T4 GPUs, with convergence typically reached in under 72 hours.

#### Single-trial cross-validation procedure

After training the model on population-level statistics as described in the previous section, we assessed generalization by splitting the correct trials into a training set (80%) and a test set (20%). The model was trained exclusively on the training set. At test time, only the trial-specific input currents $I_{i}^{\left(k\right)}$ were optimized, while all other model parameters were held fixed. All reported analyses were performed on the held-out test trials.

The rationale behind this cross-validation procedure is that the network, through its low-rank recurrent connectivity (Eq. 26), learns low-dimensional dynamics that reflect structure that is shared across trials. Therefore, when perturbed through trial-specific low-dimensional inputs, the network can reproduce individual test trials without modifying its recurrent connectivity. This demonstrates that the learned dynamics generalize across trials through low-dimensional perturbations of the network’s operating point.

#### Error axis estimation from input biases

To test whether trial-by-trial variability in residual dimensions could bias decisions, we focused on miss trials, where animals selected the incorrect lick port. For each trial, we extracted the trial-specific input biases (Eq. (25)), yielding a single feature vector in the input bias space. Correct and miss trials were then labeled, and linear classifiers (logistic regression with no intercept) were trained separately for left and right choices to distinguish correct from error trials. Performance was assessed using an 80/20 stratified split, and standard errors were estimated by bootstrap resampling (1000 iterations).

The normalized decoder weights define the error axes for left ${\vec{I}}_{\text{Error}}^{L}$ and ${\vec{I}}_{\text{Error}}^{R}$ trials, the direction in the input bias space that best separates correct from miss trials. Unlike coding and residual dimensions, which are defined in the neuronal activity space of dimension N, the error axis lies in the lower-dimensional input bias space P.

We systematically generated error trials by adding input biases to the network (Eq. (25)) along the left and right error axes.

#### Perturbations along coding and residual components of the error axes

To investigate the contribution of coding and residual dimensions to error generation, we provided the network with input biases that systematically varied in their alignment with the empirically defined error axes (${\vec{I}}_{\text{Error}}^{L}$ and ${\vec{I}}_{\text{Error}}^{R}$), ranging from purely residual to purely coding alignment. The total magnitude of each input bias was kept constant at a value sufficient to generate error-like trajectories when applied along the unmodified error axis.

To dissociate the relative contribution of coding and residual components, we continuously varied the composition of the input biases while keeping their total magnitude constant. Each input bias was constructed by linearly combining the coding and residual components of the error axis,

(31) $$\vec{I}(\alpha )={\vec{I}}_{\text{Error}}-\alpha {\vec{I}}_{\text{Error,coding}}+\alpha {\vec{I}}_{\text{Error,residual}}.$$

This interpolation spans from purely coding (α=−1) to purely residual (α=1) perturbations.

Each resulting input bias was normalized and applied to the network with the same overall magnitude, generating families of single-trial trajectories. These trajectories were then used to quantify how the coding and residual components of the left and right error axes contribute to the emergence of error-like single-trial neural dynamics (Fig. 6e-h).

### Model of ALM–Ventral Thalamus in a Memory-Guided Decision-Making Task

#### Thalamocortical loop

We extended the ALM model (Eqs. (3-11)) to include a reciprocal loop with ventral thalamus. ALM and thalamus were modeled as interacting low-rank networks coupled through dimensionality bottlenecks (see below). Based on anatomical evidence, thalamus was treated as a feedforward population: it receives cortical input via corticothalamic projections and sends output back to ALM via thalamocortical projections, without recurrent connections. The dynamics were:

(32) $$\tau_{\text{ALM}}\frac{dr_{i}^{\text{ALM}}}{dt}\;=\;-r_{i}^{\text{ALM}}+\phi_{i}\left(\sum_{j=1}^{N_{\text{ALM}}}J_{ij}^{\text{cc}}r_{j}^{\text{ALM}}+\sum_{j=1}^{N_{\text{th}}}J_{ij}^{\text{ct}}r_{j}^{\text{th}}+I_{i}^{\text{ALM}}(t)\right)$$

(33) $$\tau_{\text{th}}\frac{dr_{i}^{\text{th}}}{dt}\;=\;-r_{i}^{\text{th}}+\phi_{i}\left(\sum_{j=1}^{N_{\text{ALM}}}J_{ij}^{\text{tc}}r_{j}^{\text{ALM}}+I_{i}^{\text{th}}(t)\right)$$

Here, $J^{\text{cc}}$ denotes recurrent cortical connectivity, $J^{\text{ct}}$ the thalamocortical projections, and $J^{\text{tc}}$ the corticothalamic projections. In line with experimental evidence, the thalamic integration time constant was set to one third of the cortical value ($\tau_{\text{th}}=\tau_{\text{ALM}}∕3=40∕3\;\text{ms}$). Consistent with anatomical data, no recurrent connectivity was included within thalamus.

#### ALM local connectivity

ALM connectivity was written similarly as in Eq. (5),

(34) $$J_{ij}^{\text{cc}}=\sum_{l=1}^{P_{\text{ALM}}}\sum_{l^{\prime }=1}^{P_{\text{ALM}}}A_{ll^{\prime }}^{\text{cc}}u_{i}^{l,\text{ALM}}u_{j}^{l^{\prime },\text{ALM}},$$

with $A^{\text{cc}}$ trainable.

#### Thalamocortical and corticothalamic connectivity

We modeled thalamocortical and corticothalamic projections as operating through low-dimensional bottlenecks in latent space (Barbosa et al. 2023; Clark & Beiran 2024; Pereira-Obilinovic et al. 2024). This reflects the idea that only a restricted set of activity patterns is transmitted between ALM and thalamus, while the remaining dimensions remain local, as shown previously for cortico-cortical communication (Semedo et al. 2019; MacDowell et al. 2025).

In the model, each projection was written as a product of three matrices: a readout from the presynaptic subspace, a bottleneck that mixes signals in a reduced set of dimensions, and a projection into the postsynaptic subspace (see Fig. 7b):

(35) $$J^{\text{ct}}=U^{\text{ALM}}J^{\text{ct,post}}B^{\text{ct}}J^{\text{ct,pre}}{\left(U^{\text{th}}\right)}^{T},\;J^{\text{tc}}=U^{\text{th}}J^{\text{tc,post}}B^{\text{tc}}J^{\text{tc,pre}}{\left(U^{\text{ALM}}\right)}^{T}.$$

Here $U^{\text{ALM}}$ and $U^{\text{th}}$ span the coding and residual subspaces in ALM (see Eq. (34)) and thalamus, respectively. For the thalamocortical projections, $J^{\text{ct,pre}}$ selects a $P^{\text{ct}}$-dimensional subspace of the thalamic latent space ($P^{\text{ct}}\leq P^{\text{th}}$). These signals are mixed within the bottleneck $B^{\text{ct}}$ and projected into a $P^{\text{ct}}$-dimensional subspace of ALM defined by $J^{\text{ct},\text{post}}(P^{\text{ct}}\leq P^{\text{ALM}})$.

Analogously, corticothalamic communication is restricted to a $P^{\text{tc}}$-dimensional subspace of ALM selected by $J^{\text{tc,pre}}$, transformed through $B^{\text{tc}}$, and projected into thalamus by $J^{\text{tc,post}}$.

Thus, despite the higher dimensionality of both ALM and thalamus, reciprocal communication is limited to two narrow subspaces: $P^{\text{ct}}$ thalamic dimensions transmitted to ALM and $P^{\text{tc}}$ ALM dimensions transmitted to thalamus. The remaining dimensions in each area remain private and do not propagate across the loop.

For Fig. 7, we set the latent dimensionality of ALM and thalamus to $P^{\text{ALM}}=P^{\text{th}}=10$, with both the thalamocortical and corticothalamic bottlenecks restricted to $P^{\text{ct}}=P^{\text{tc}}=5$.

Lastly, the external inputs to the network followed the same structure as described in Eqs.(6-10), with one key difference: consistent with experimental findings (Inagaki et al. 2022), the go cue was delivered to the thalamic network, whereas the sample stimulus was delivered to ALM.

#### Training procedure

Training followed the same procedure described for the cortical model described previously, but was extended to include both ALM and thalamus. The reconstruction loss was computed jointly across the two areas, requiring the model to reproduce condition-averaged activity in both ALM and thalamus. Coding alignment losses were applied separately in each area, constraining the learned low-rank subspaces ($U^{\text{ALM}}$, $U^{\text{th}}$) so that their coding dimensions aligned with task-relevant axes extracted from ALM or thalamic recordings. Orthogonality losses were likewise imposed independently within ALM and thalamus. The total loss was a weighted combination of reconstruction, alignment, and orthogonality terms, using the same coefficients as in the cortical model. Models were trained separately for left and right hemispheres with the Adam optimizer (learning rate 5 × 10^−4^) and gradient clipping.

#### Random perturbations in thalamus

In Fig. 7e, following the perturbation analysis described above (see Network perturbations), we tested the sensitivity of the thalamocortical model to random inputs applied to thalamus. Perturbations were of the form

(36) $$I_{\text{pert}}=g\vec{p},$$

where g is the perturbation magnitude and $\vec{p}$ is either the thalamic choice axis or a random direction within the residual subspace. For 40 magnitudes (linearly spaced between 1 and 6), we applied inputs along the choice axis or along 120 random residual directions. Random vectors were sampled from a standard normal distribution and normalized to unit norm.

The effect of each perturbation was quantified as the mean squared reconstruction error between perturbed model activity and condition-averaged neural activity in ALM and thalamus. Losses were compared across choice-axis and residual-subspace perturbations to assess the relative influence of task-relevant versus residual dimensions.

## Supplementary Material

1
