# Supplementary material for: Neural dynamics outside task-coding dimensions drive decision trajectories through transient amplification
Source: bioRxiv. 2025 Nov 22:2025.11.20.689599. Preprint. [Version 1] doi: 10.1101/2025.11.20.689599 (PMC12667970; doi:10.1101/2025.11.20.689599)
Supplement: 1 [file NIHPP2025.11.20.689599v1-supplement-1.pdf]

bioRxiv preprint doi: https://doi.org/10.1101/2025.11.20.689599; this version posted November 22, 2025. The copyright holder for this preprint (which was not certified by peer review) is the author/funder, who has granted bioRxiv a license to display the preprint in perpetuity. It is made available under aCC-BY 4.0 International license.

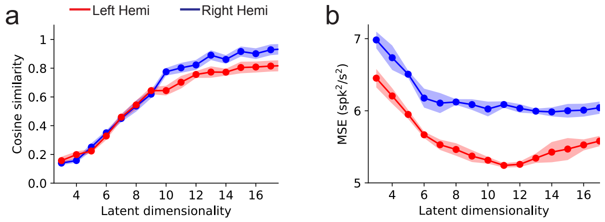

Figure S1: Supplementary Figure corresponding to Fig. 1. (a) Average cosine similarity between the coding dimensions extracted from the network fit and those derived from the data, computed across five independent subsets using 80% of the trials to estimate trial-averaged activity and train the network, shown as a function of model dimensionality. (b) Cross-validated mean squared error (MSE) between the network's firing rates and the trial-averaged activity computed on the held-out 20% of trials in each subset. Error bars indicate the standard error across folds (see Methods). Red traces correspond to networks trained on data from the left hemisphere, while blue traces correspond to networks trained on data from the right hemisphere.

bioRxiv preprint doi: https://doi.org/10.1101/2025.11.20.689599; this version posted November 22, 2025. The copyright holder for this preprint (which was not certified by peer review) is the author/funder, who has granted bioRxiv a license to display the preprint in perpetuity. It is made available under aCC-BY 4.0 International license.

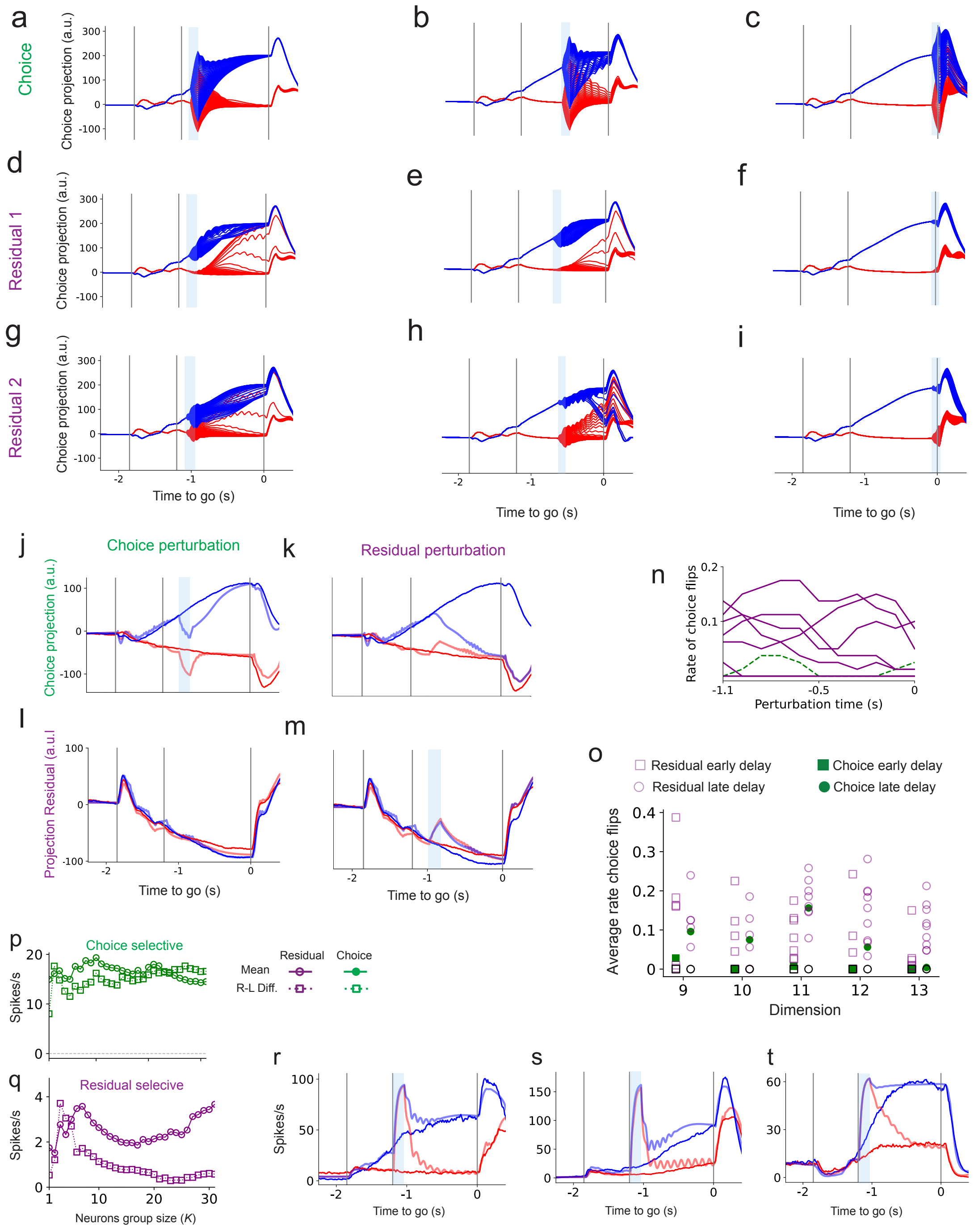

Figure S2: Supplementary Figure corresponding to Fig. 2. Projections onto the choice dimension for perturbations aligned with the choice dimension (a–c), the influential residual dimension from Fig. 2d,f (d–f), and the influential residual dimension from Fig. 2i (g–i), shown at the beginning, middle, and end of the delay period. All data in panels (a–i) correspond to the same network from the **Right Hemisphere** shown in Fig. 2 ($P = 10$). (j–m) Projections onto the choice (j,k) and residual (l,m) dimensions for perturbations aligned with the choice and residual directions, respectively, for a *different network trained with data from the* **Left Hemisphere**. The blue bar indicates the perturbation period. (n) Rate of choice flips for 11 distinct 100 ms perturbation intervals during the delay period across 10 network dimensions, using perturbation magnitudes from –15 to 15, for the Left Hemisphere network. (o) Extension of Fig. 2g showing average choice-flip rates from Fig. 2h, split into early (–1.1 to –0.6 s) and late (–0.5 to 0 s) delay periods, summarized across networks trained with 9 to 13 dimensions. (p–q) Solid and circles: mean firing rate averaged over the delay period and over the top-K selective neurons, pooling left and right trials. Dashed and squares: mean right–minus–left firing-rate difference, averaged over the same delay window and neuron set. (p) choice-selective neurons; (q) residual-selective neurons. (r–t) Responses of the top three choice-selective neurons to perturbations targeting the top 30 choice-selective neurons using a positive perturbation (same magnitude and opposite sign to Fig. 2k).

bioRxiv preprint doi: https://doi.org/10.1101/2025.11.20.689599; this version posted November 22, 2025. The copyright holder for this preprint (which was not certified by peer review) is the author/funder, who has granted bioRxiv a license to display the preprint in perpetuity. It is made available under aCC-BY 4.0 International license.

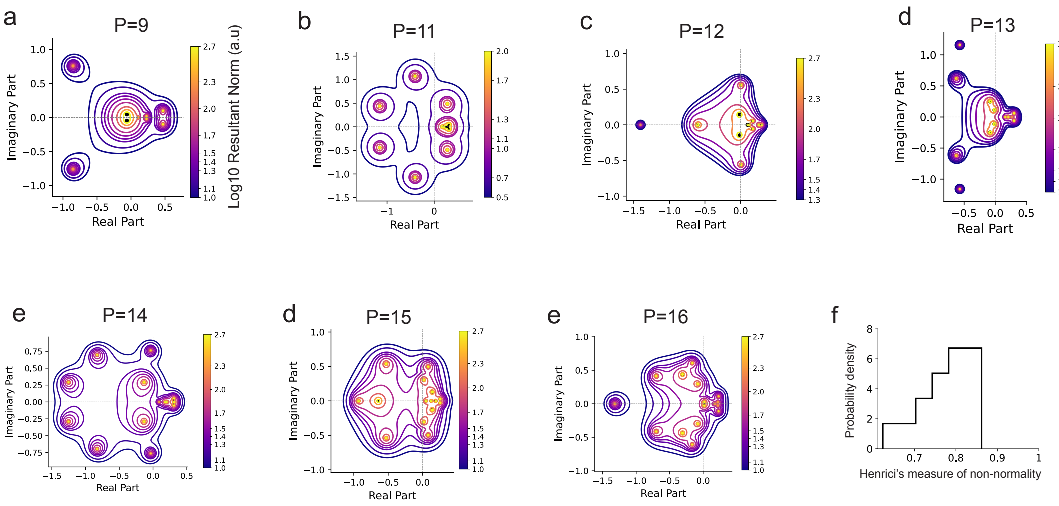

Figure S3: Supplementary Figure corresponding to Fig. 3. Eigenvalue spectra and pseudospectra of the interaction matrix (Trefethen & Embree 2020) for networks with dimensionality $P = 9$ (a) and $P = 11–16$ (b–e). (f) Normalized Henrici index (see Methods) across 15 trained networks with latent dimensionality ranging from P = 9 to P = 16 trained on neurons recorded on the left hemisphere.

bioRxiv preprint doi: https://doi.org/10.1101/2025.11.20.689599; this version posted November 22, 2025. The copyright holder for this preprint (which was not certified by peer review) is the author/funder, who has granted bioRxiv a license to display the preprint in perpetuity. It is made available under aCC-BY 4.0 International license.

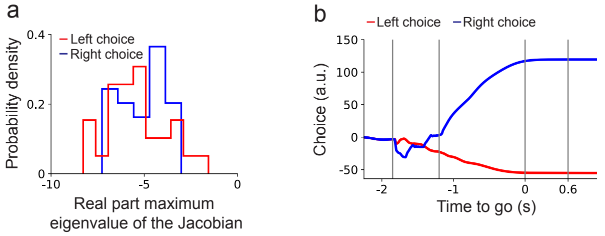

Figure S4: Supplementary Figure corresponding to Fig. 4. (a) Distribution over trained networks of the real part of the eigenvalue with the largest real part from the Jacobian matrix, computed at the choice-selective fixed points across 48 trained networks with latent dimensionality $P = 9$–16. Negative values indicate dynamical stability. (b) Projection onto the choice dimension for a network trained on ALM recordings from the left hemisphere ($P = 13$) when the go cue is withheld, illustrating convergence to a stable choice-selective attractors.

bioRxiv preprint doi: https://doi.org/10.1101/2025.11.20.689599; this version posted November 22, 2025. The copyright holder for this preprint (which was not certified by peer review) is the author/funder, who has granted bioRxiv a license to display the preprint in perpetuity. It is made available under aCC-BY 4.0 International license.

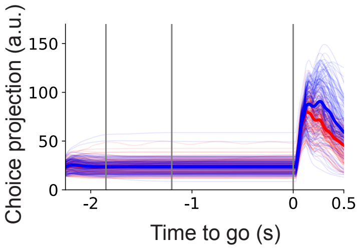

Figure S5: Supplementary Figure corresponding to Fig. 5. When the sample stimulus is withheld, the network fails to sustain delay-period activity, indicating that integration of the sample input is required to generate the preparatory activity. Input biases alone cannot account for the data, choice-encoding delay activity emerges only when the network integrates the sample stimulus.

bioRxiv preprint doi: https://doi.org/10.1101/2025.11.20.689599; this version posted November 22, 2025. The copyright holder for this preprint (which was not certified by peer review) is the author/funder, who has granted bioRxiv a license to display the preprint in perpetuity. It is made available under aCC-BY 4.0 International license.

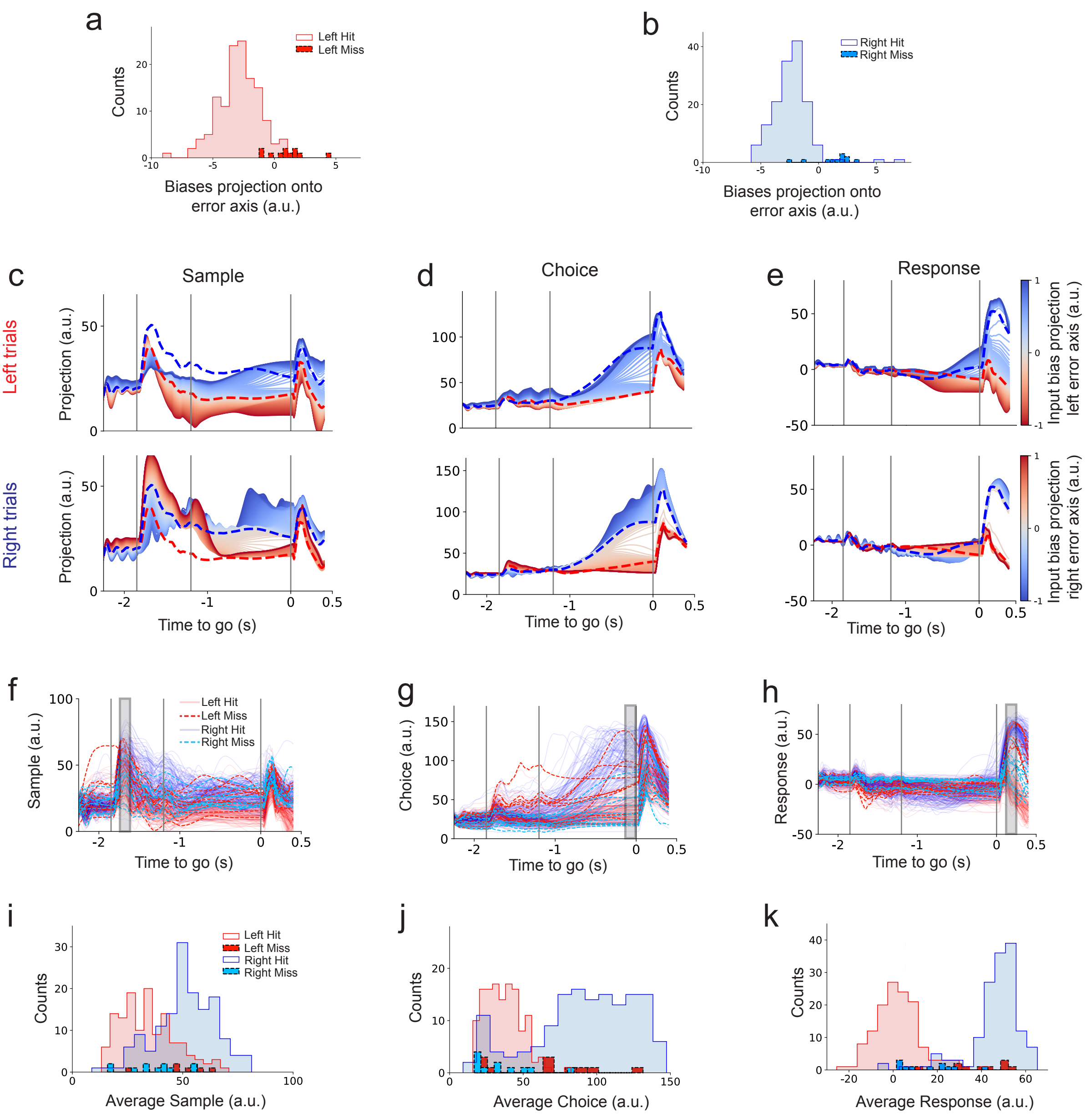

Figure S6: Supplementary Figure corresponding to Fig. 6. (a, b) Projections of input biases onto the left (a) and right (b) decoder weights (error axes) for hit and miss trials in left (a) and right (b) conditions. (c–e) Network projections onto stimulus, choice, and response dimensions following perturbations along the error axes. Top: left stimulus trials; bottom: right stimulus trials. (f–e) Single-trial network trajectories projected onto stimulus, choice, and response dimensions for left (red) and right (blue) trials. Solid lines: hit trials; dashed lines: miss trials. Deep red and cyan indicate left and right miss trials, respectively. (i–k) Histograms of time-averaged projections (100 ms window; gray rectangles in f–h) across stimulus, choice, and response dimensions for each trial type.

bioRxiv preprint doi: https://doi.org/10.1101/2025.11.20.689599; this version posted November 22, 2025. The copyright holder for this preprint (which was not certified by peer review) is the author/funder, who has granted bioRxiv a license to display the preprint in perpetuity. It is made available under aCC-BY 4.0 International license.

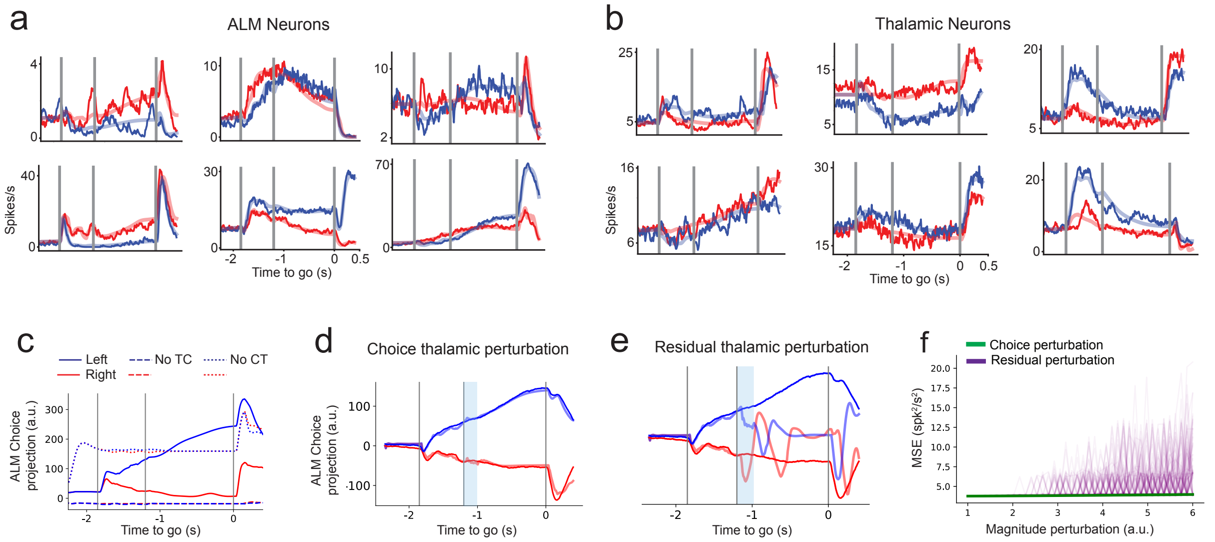

Figure S7: Supplementary Figure corresponding to Fig. 7. (a–b) Trial-averaged activity for correct left and right trials. Solid traces show neural recordings; lighter traces show model fits. Data are from the left hemisphere. Both cortex and thalamus were modeled with latent dimensionality $P = 10$, and both thalamocortical and corticothalamic projections were constrained to a bottleneck of 5 dimensions (see Methods). (a) ALM neurons; (b) VM and VL thalamic neurons. (c) Choice projection in ALM for three conditions: unperturbed (solid), thalamocortical projections removed (No TC, dashed), and corticothalamic projections removed (No CT, dotted). (d–e) Choice projection following perturbations in thalamus along the choice dimension (d) and a residual dimension (e). Yellow bar indicates the perturbation period. (f) Mean squared error (MSE) between unperturbed and perturbed trajectories for 120 perturbations along residual (purple) and choice (green) dimensions at increasing magnitudes. Light traces show individual perturbations; solid lines show the average. Panels (d–f) are from the left hemisphere and correspond to Fig. 7c–e.
